# Improvement in health-related quality of life after treatment with resmetirom in patients with the spectrum of MASLD: From early MASH to MASH cirrhosis

**DOI:** 10.1097/HC9.0000000000000913

**Published:** 2026-02-26

**Authors:** Zobair M. Younossi, Fatema Nader, Dominic Labriola, Rebecca Taub, Andrei Racila, Linda Henry, Maria Stepanova

**Affiliations:** 1 The Global NASH/MASH Council, Washington, District of Columbia, USA; 2 Beatty Liver and Obesity Research Program, Inova Health System, Falls Church, Virginia, USA; 3 Madrigal Pharmaceuticals, West Conshohocken, Pennsylvania, USA; 4 Center for Outcomes Research in Liver Diseases, Washington, District of Columbia, USA

**Keywords:** abdominal symptoms, health distress, hepatic steatosis, patient-reported outcomes, Worry

## Abstract

**Background:**

Resmetirom is approved for MASH F2–F3 fibrosis. Health-related quality of life (HRQL) was assessed in patients with MASH treated with resmetirom.

**Methods:**

Patients with MASH enrolled in MAESTRO-NAFLD-1 (NCT04197479) and the 52-week open-label extension study (year 2). HRQL was assessed using LDQoL and CLDQ-NAFLD. Magnetic resonance imaging–proton density fat fraction (MRI-PDFF) response definition for early MASH: a decrease ≥30% from baseline; MASH cirrhosis ≥20% decrease (if baseline MRI-PDFF >5%).

**Results:**

MAESTRO-NAFLD-1 included 180 patients with MASH cirrhosis and 1143 with early MASH [baseline MRI-PDFF ≥8%, vibration-controlled transient elastography (VCTE) ≥5.5 kPa <8.5 kPa] treated with 80 mg or 100 mg resmetirom or placebo or 100 mg open-label resmetirom. Baseline HRQL for MASH cirrhosis was significantly lower (up to −15% of score range) for all 6 domains of CLDQ-NAFLD and 13/17 domains of LDQoL (*p*<0.05). MASH cirrhosis, by week 24 of resmetirom treatment year 1, had improvements in Worry (CLDQ-NAFLD) and Health Distress (LDQoL) up to +4% of score range, sustained through week 52 and throughout year 2 (day 1, week 12, week 28) (*p*<0.05). Cirrhotic patients with MRI-PDFF response by year 1 week 52, improved in Stigma score (LDQoL): mean change (+3.9 vs. −4.2, *p*=0.042). Early MASH receiving resmetirom experienced improvement in: Abdominal and Worry (CLDQ-NAFLD) and Health Distress (LDQoL) (up to +5% of score range size); no similar improvements observed in placebo (all *p*>0.05). Early MASH patients with MRI-PDFF response experienced Abdominal and Worry score (up to +6%) improvements—greater than placebo or nonresponders.

**Conclusions:**

Early MASH and MASH cirrhosis treated with resmetirom experience improvement in some disease-specific HRQL scores.

## Graphical abstract

**Figure:**
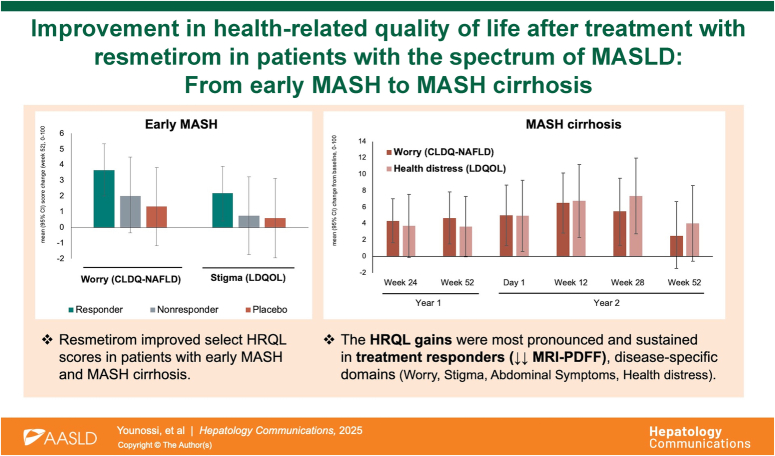

## INTRODUCTION

Over the past 2 decades, metabolic dysfunction–associated steatotic liver disease (MASLD), formerly known as nonalcoholic fatty liver disease (NAFLD), has emerged as one of the most prevalent causes of chronic liver disease worldwide.^1^,^2^,^3^ MASLD now ranks among the top 2 leading causes of cirrhosis, hepatocellular carcinoma (HCC), and liver transplantation.^4^,^5^,^6^,^7^ This rise mirrors the global epidemics of obesity and type 2 diabetes (T2D), both of which are closely linked to the development and progression of MASLD.^8^,^9^,^10^ As the global burden of MASLD continues to increase, it presents a significant clinical and public health challenge.

Until recently, the treatment of MASLD has relied solely on lifestyle interventions. While effective in some patients, lifestyle modifications are difficult to achieve and maintain, yielding suboptimal results, particularly in individuals with more advanced disease.^11^ Given that, pharmacologic therapies that directly target the underlying pathophysiology of MASLD have long been awaited. Resmetirom, a selective thyroid hormone receptor-β (THR-β) agonist, represents a novel therapeutic approach designed to reduce hepatic steatosis, inflammation, and fibrosis without the adverse effects associated with non-selective thyroid hormone activity.^12^ In 2024, resmetirom became the first drug approved in the United States for the treatment of non-cirrhotic metabolic dysfunction–associated steatohepatitis (MASH) with moderate to advanced fibrosis (stages F2–F3); in 2025, resmetirom was also approved in Europe. Beyond histologic improvement, it has been shown that resmetirom improves select domains of health-related quality of life (HRQL) in patients with biopsy-proven MASH and significant fibrosis.^13^ However, the impact of resmetirom on HRQL across the broader MASLD population, including those with cirrhosis, remains unclear.

The aim of this analysis was to evaluate the effects of resmetirom on HRQL in a diverse cohort of patients with MASLD, including both non-cirrhotic and cirrhotic individuals, who were enrolled in the phase 3 MAESTRO-NAFLD clinical trials.

## METHODS

The study presents a pre-specified analysis of HRQL data collected in a 52-week phase 3, randomized, double-blind, placebo-controlled study of resmetirom in subjects with suspected or confirmed MASH or MASLD (presumed MASH) (MAESTRO-NAFLD-1, NCT04197479).^14^ The study was conducted before the change in nomenclature was finalized and originally used the terms NAFLD and NASH; the respective diagnoses were subsequently renamed as MASLD and MASH.

The main study inclusion criteria were: liver stiffness measurement (LSM) by vibration-controlled transient elastography (VCTE) ≥5.5 kPa to <8.5 kPa with CAP ≥280 dB/m, or MRE ≥2.0 to <4.0 kPa with magnetic resonance imaging–proton density fat fraction (MRI-PDFF) ≥8%, or if a recent liver biopsy, fibrosis stage ≤3. Patients with VCTE ≥8.5 kPa were eligible at sites that were not recruiting for MAESTRO-NASH^12^ or in patients who had screen-failed MAESTRO-NASH based on a low NASH score or fibrosis stage of 1. Based on a VCTE threshold of ≥7.2 kPa, approximately one-third of patients were classified as stage F2, while the remaining two-thirds were stage F1; therefore, this sample is referred to as early MASH throughout this manuscript. In addition to 3 double-blind arms (resmetirom 100 mg, resmetirom 80 mg, placebo), early MASH patients could have been assigned to open-label treatment with resmetirom 100 mg. The study also included a group of patients with well-compensated MASH cirrhosis without evidence of or history of decompensation. To be eligible, patients must have had well-compensated MASH cirrhosis diagnosed by liver biopsy (~60%), a historic biopsy with MASH F2–F3 fibrosis and progression to MASH cirrhosis, or MASH cirrhosis based on clinical and laboratory parameters. Full eligibility criteria have been published elsewhere.^14^

Per the MAESTRO-NAFLD-1 protocol,^14^ early MASH patients were randomized in a 1:1:1:1 ratio between the 4 treatment arms: double-blind resmetirom 100 mg, resmetirom 80 mg, matching placebo, or open-label resmetirom 100 mg; randomization was switched to a 1:1:1 ratio between 3 treatment arms (double-blind resmetirom 100 mg, resmetirom 80 mg, or matching placebo) after a pre-specified number of patients were enrolled in the open-label arm. The randomization was stratified by type 2 diabetes status (presence/absence) and documented atherosclerotic cardiovascular disease (ASCVD). Subjects with MASH cirrhosis were assigned to receive 80 mg of resmetirom once daily.

For early MASH, MRI-PDFF response was defined as a decrease in MRI-PDFF ≥30% from baseline; an alternative cutoff of ≥50% was also evaluated. For MASH cirrhosis, MRI-PDFF response was defined as ≥20% decrease in MRI-PDFF (only in those with baseline MRI-PDFF >5%). In addition, for patients with MASH cirrhosis, VCTE response was defined as a ≥25% decrease in LSM by VCTE.

### Health-related quality of life

The HRQL was assessed using the Chronic Liver Disease Questionnaire (CLDQ-NAFLD) and Liver Disease Quality of Life (LDQoL) instruments. The instruments were completed by all subjects on the first day of treatment before the initiation of any treatment-related activities, then at week 24 and week 52 of treatment.

The CLDQ-NAFLD is a validated NAFLD/NASH-specific instrument that includes 36 items and 6 domains (Abdominal symptoms, Activity, Emotional health, Fatigue, Systemic symptoms, Worry).^15^ All items and domain scores range from 1 to 7; higher scores indicate better quality of life. In this manuscript, where specified, the CLDQ-NAFLD scores were renormalized to 0–100 for illustrative purposes.

The LDQOL is a two-part HRQL instrument which includes 72 items and 17 domains: the first 36 items and 8 domains are generic (Physical Functioning, Role Physical, Bodily Pain, General Health, Vitality, Social Functioning, Role Emotional, Mental Health), and the remaining 36 items and 9 domains are liver disease-specific (Symptoms of liver disease, Effects of liver disease, Concentration/memory, Health distress, Sexual Function, Sleep, Loneliness, Hopelessness, and Stigma of liver disease).^16^ All domain scores range from 0 to 100; higher scores indicate better quality of life.

For both HRQL instruments, the minimal clinically important difference (MCID) in a score was defined as 5% of the score range size, that is, 0.3 for the domains of CLDQ-NAFLD, and 5.0 for the domains of LDQoL.

### Statistical analysis

The sample size for the MAESTRO-NAFLD-1 study was determined to provide sufficient power for assessing the study’s key endpoint (the percent change in LDL-C),^14^ without consideration of the present HRQL analysis. The HRQL scores were summarized as N (%) or mean±standard deviation, in patients with early MASH and with cirrhosis separately. Only observed HRQL data at each study time point were used.

In subjects with early MASH, the mixed-effects models for repeated measures (MMRMs) that included both week 24 and week 52 HRQL measurements were used to estimate the effect of treatment regimen on changes in HRQL scores from subjects’ own baseline levels, for each HRQL domain separately. The MMRMs included adjustment for the baseline HRQL value and stratification factors (the presence of diabetes and ASCVD), treatment regimen as a fixed effect, and subject as a random effect, and yielded least-square mean (LSM) estimates for the HRQL score changes at week 24 and week 52 with 97.5% confidence intervals. The effect of treatment in early MASH subjects was evaluated in 2 steps: first, 100 mg double-blind versus 100 mg open-label; second (if no significant effect is observed at the prior step), pooled 100 mg (double-blind and open-label) and 80 mg versus placebo. Assessments of changes in HRQL scores in other pre-defined clinical groups (by the presence of treatment response) and in patients with MASH cirrhosis were performed via arithmetic means, which were compared between the subgroups using the Mann–Whitney test, and to zero (which would indicate no significant change from baseline) using the Wilcoxon signed-rank test for matched pairs. In addition, the proportions of subjects meeting MCID were calculated.

All analyses were conducted in SAS 9.4 (SAS Institute, Cary, NC). All participants provided written informed consent before enrollment. This study was done in accordance with the ethical principles of the Declarations of Helsinki and Istanbul and was consistent with the International Conference on Harmonisation Good Clinical Practice and applicable regulatory requirements. The institutional review boards or independent ethics committee of each study center approved the study and all amendments.

## RESULTS

### HRQL in early MASH

There were 1143 early MASH patients included: 325 received double-blind resmetirom 100 mg, 327 double-blind resmetirom 80 mg, 320 placebo, and 171 open-label treatment with resmetirom 100 mg (Supplemental Table S1, http://links.lww.com/HC9/C262). The mean (±SD) age was 56±12 years, 43% were male, 53% had type 2 diabetes, and the mean baseline MRI-PDFF was 18±7%; more detailed clinical presentation and outcomes of the MAESTRO-NAFLD-1 study have been published elsewhere.^14^

At baseline, patients with early MASH reported some impairment in HRQL, with mean CLDQ-NAFLD domain scores ranging from 4.9 in Fatigue to 6.1 in Worry (out of the maximum of 7) (Supplemental Table S1, http://links.lww.com/HC9/C262). The LDQoL scores showed more variation across domains, with relatively high values in areas such as Role Emotional (mean score 87), Social Functioning (85), and Health Distress (89), and lower scores in Sleep (65) and Vitality (60). These values were consistent across the randomized treatment arms and the open-label cohort (Supplemental Table S1, http://links.lww.com/HC9/C262).

Weak or no correlations were observed between baseline HRQL scores and baseline MRI-PDFF. Statistically significant were correlations of baseline MRI-PDFF with General Health (ρ=−0.06), Vitality (ρ=−0.10), Mental Component Summary (ρ=−0.06), and Stigma of liver disease (ρ=−0.07) (*p*<0.05) (Supplemental Table S2, http://links.lww.com/HC9/C262).

The HRQL changes from baseline to week 24 or week 52 of double-blind treatment with 100 mg resmetirom were not different from those observed in the open-label group (all *p*>0.05) (Supplemental Table S3, http://links.lww.com/HC9/C262). Therefore, for all further analyses, the double-blind 100 mg and open-label 100 mg groups will be pooled.

By weeks 24 and 52 of treatment with resmetirom, early MASH patients receiving placebo experienced a statistically significant worsening of HRQL in some physical health-related domains of the LDQoL, including Role physical and Vitality: mean decreases of up to −5.5% (*p*<0.05) (Supplemental Table S4, http://links.lww.com/HC9/C262). In addition, at week 24, the Worry domain of CLDQ-NAFLD improved significantly with resmetirom (mean +0.17 for 100 mg, +0.16 for 80 mg, both *p*<0.05) compared with placebo (mean +0.04, *p*>0.05); the improvements in the Abdominal symptoms domain of CLDQ-NAFLD were also observed as follows: mean +0.15 in 100 mg (*p*<0.05), +0.14 in 80 mg, +0.10 in placebo (Supplemental Table S4, http://links.lww.com/HC9/C262). By week 52, Abdominal symptoms scores improved significantly with both resmetirom doses (both +0.28, *p*<0.05) while placebo showed minimal change (+0.05, *p*>0.05), and improvements in the Worry domain scores sustained (+0.15 in 100 mg, +0.22 in 80 mg, both *p*<0.05, vs. +0.04 in placebo) (Supplemental Table S4, http://links.lww.com/HC9/C262). The domain of Health distress of LDQoL also improved in patients receiving resmetirom: +1.33 in 100 mg, +2.36 in 80 mg vs. −1.25 in placebo (*p*<0.05) at week 24; +2.07 in 100 mg, +3.25 in 80 mg vs. +0.09 in placebo (*p*<0.05) at week 52 (Supplemental Table S4, http://links.lww.com/HC9/C262). In addition, resmetirom-treated patients experienced smaller declines in Role physical and Role emotional scores of LDQoL in comparison to placebo (Supplemental Table S4, http://links.lww.com/HC9/C262). The proportions of patients meeting MCID for each HRQL domain in the resmetirom-treated groups and placebo at each treatment time point are shown in Supplemental Table S5, http://links.lww.com/HC9/C262.

**FIGURE 1:**
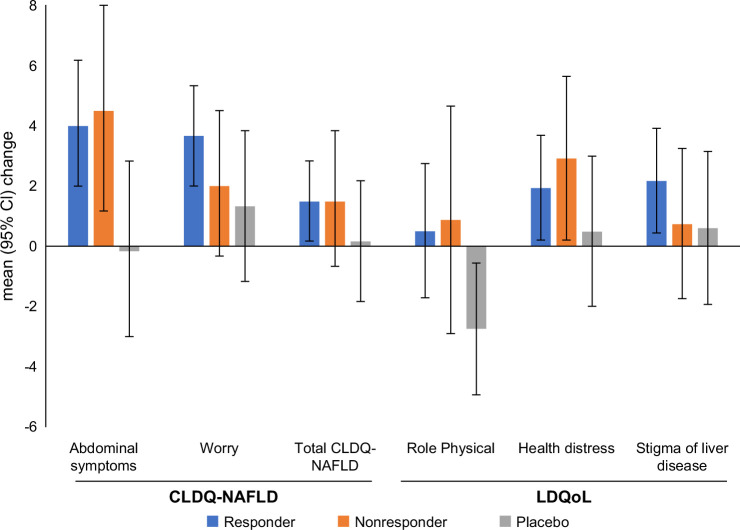
Changes in HRQL scores in early MASH patients with (Responder) versus without MRI-PDFF (≥30%) response (Nonresponder) to treatment with resmetirom versus placebo; mean HRQL score change (renormalized from the original scale to 0–100 where applicable) from baseline to week 52 with 95% CI. Only statistically significant changes from baseline to week 52 (two-sided *p*<0.05 in comparison to zero or to nonresponders or to placebo) are shown; changes in all HRQL domains in their original scales are shown in Supplemental Figure S1, http://links.lww.com/HC9/C262. Abbreviations: CLDQ-NAFLD, Chronic Liver Disease Questionnaire–Nonalcoholic Fatty Liver Disease; HRQL, health-related quality of life; LDQoL, Liver Disease Quality of Life; MASH, metabolic dysfunction–associated steatohepatitis; MRI-PDFF, magnetic resonance imaging–proton density fat fraction.

Patients who achieved ≥30% reduction in MRI-PDFF from baseline by week 52 experienced generally greater improvements in select HRQL scores in comparison to placebo or to nonresponders (Figure 1 and Supplementary Figure 1). Improvements in Abdominal symptoms (mean +0.18 in 100 mg, +0.38 in 80 mg vs. −0.01 in placebo), Worry (+0.21 in 100 mg, +0.24 in 80 mg vs. +0.08 in placebo), and total CLDQ-NAFLD (+0.15 in 80 mg vs. +0.01 in placebo) were statistically significant (*p*<0.05) (Table 1 and Supplemental Table S6, http://links.lww.com/HC9/C262). Despite this, patients with MRI-PDFF response experienced some decline in their LDQoL scores (Effects of liver disease, Sleep), which, however, were similar or smaller than those seen in placebo (Table 1 and Supplemental Table S6, http://links.lww.com/HC9/C262). These patterns sustained with the ≥50% MRI-PDFF reduction threshold, with additional improvements observed in Vitality (mean +2.75 in 100 mg vs. −1.77 in placebo, *p*<0.05), Stigma (+3.21 in 100 mg vs. +0.60 in placebo, *p*<0.05), and identical trends in the liver-specific domains of Abdominal symptoms and Worry of CLDQ-NAFLD (Table 1 and Supplemental Table S6, http://links.lww.com/HC9/C262).

**TABLE 1:** Changes in HRQL scores in resmetirom-treated patients with early MASH with MRI-PDFF response versus placebo

MRI-PDFF response (30%)	Response with resmetirom 100 mg	Response with resmetirom 80 mg	Placebo
Abdominal symptoms	0.18 (0.02–0.33)^a^	0.38 (0.17–0.59)^a^ ^b^	−0.01 (−0.18 to 0.17)
Worry	0.21 (0.09–0.34)^a^	0.24 (0.08–0.39)^a^	0.08 (−0.07 to 0.23)
Total CLDQ-NAFLD score	0.06 (−0.04 to 0.16)	0.15 (0.02–0.29)^a^	0.01 (−0.11 to 0.13)
Role Physical	1.05 (−1.72 to 3.81)^b^	−0.53 (−4.26 to 3.19)	−2.74 (−4.93 to −0.56)^a^
Vitality	0.68 (−1.61 to 2.96)	1.07 (−1.87 to 4.01)	−1.77 (−3.75 to 0.20)^a^
Role Emotional	−0.45 (−3.18 to 2.27)	−0.78 (−4.03 to 2.46)	−2.81 (−5.02 to −0.60)^a^
Mental Component summary	−0.30 (−1.39 to 0.80)	0.05 (−1.12 to 1.23)	−0.89 (−1.86 to 0.09)^a^
Effects of liver disease	−1.37 (−3.03 to 0.29)	−3.08 (−5.47 to −0.68)^a^	−1.90 (−3.65 to −0.15)^a^
Concentration/memory	−1.58 (−3.77 to 0.61)	−2.35 (−4.76 to 0.06)	−3.23 (−5.35 to −1.12)^a^
Sleep	−2.36 (−4.60 to −0.13)^a^	−0.47 (−3.24 to 2.30)	−0.92 (−3.04 to 1.20)
Stigma of liver disease	2.17 (0.04-4.31)^a^	2.19 (−0.81 to 5.19)	0.60 (−1.93 to 3.14)
MRI-PDFF response (50%)			
Abdominal symptoms	0.18 (−0.01 to 0.37)	0.36 (0.08–0.63)^a^ ^b^	−0.01 (−0.18 to 0.17)
Worry	0.20 (0.05–0.35)^a^	0.16 (−0.03 to 0.36)	0.08 (−0.07 to 0.23)
Role Physical	1.86 (−1.16 to 4.88)^b^	−1.87 (−6.51 to 2.77)	−2.74 (−4.93 to −0.56)^a^
Vitality	2.75 (0.12–5.38)^a^ ^b^	0.41 (−3.18 to 3.99)	−1.77 (−3.75 to 0.20)^a^
Role Emotional	−1.03 (−3.99 to 1.94)	−3.68 (−6.71 to −0.65)^a^	−2.81 (−5.02 to −0.60)^a^
Physical Component Summary	0.87 (−0.12 to 1.87)^a^	−0.01 (−1.41 to 1.39)	0.01 (−0.77 to 0.80)
Mental Component Summary	−0.25 (−1.48 to 0.98)	−0.77 (−1.96 to 0.41)	−0.89 (−1.86 to 0.09)^a^
Effects of liver disease	−2.11 (−3.95 to −0.27)^a^	−2.77 (−5.25 to −0.29)^a^	−1.90 (−3.65 to −0.15)^a^
Concentration/memory	−2.26 (−4.89 to 0.37)	−3.65 (−6.67 to −0.64)^a^	−3.23 (−5.35 to −1.12)^a^
Sleep	−2.68 (−5.27 to −0.09)^a^	−0.52 (−3.93 to 2.89)	−0.92 (−3.04 to 1.20)
Stigma of liver disease	3.21 (0.66 to 5.77)^a^	0.49 (−3.56 to 4.54)	0.60 (−1.93 to 3.14)

Each cell shows the mean change from baseline to week 52 with 95% CI.

^a^ Two-sided *p*<0.05 versus zero change (within-treatment comparison, indicates significant change from baseline).

^b^ Two-sided *p*<0.05 versus placebo. Only statistically significant changes (two-sided *p*<0.05 in comparison to zero or to placebo) are included; all HRQL domains are shown in Supplemental Table S6, http://links.lww.com/HC9/C262.

Abbreviations: CLDQ-NAFLD, Chronic Liver Disease Questionnaire–nonalcoholic fatty liver disease; HRQL, health-related quality of life; MASH, metabolic dysfunction–associated steatohepatitis; MRI-PDFF, magnetic resonance imaging–proton density fat fraction.

When combining both resmetirom doses, pooled MRI-PDFF treatment responders (with the ≥30% MRI-PDFF reduction cutoff) had significantly greater improvements in the Abdominal symptoms (+0.24 vs. −0.01), Worry (+0.22 vs. +0.08), Health distress (+1.94 vs. +0.49), and Stigma of liver disease (+2.18 vs. +0.60) scores when compared with placebo or to zero (*p*<0.05) (Figure 1, Table 2, and Supplemental Table S7, http://links.lww.com/HC9/C262). Responders at the ≥50% threshold showed additional incremental benefits in Role Physical (+0.68 vs. −2.74) and Vitality (+1.94 vs −1.63) (Table 2 and Supplemental Table S7, http://links.lww.com/HC9/C262). The decreases in select LDQoL scores (Effects of liver disease, Concentration/memory, Sleep) were similar to those seen in placebo (all *p*>0.05) (Table 2 and Supplemental Table S7, http://links.lww.com/HC9/C262). In MCID-based analyses, Vitality showed consistently greater MCID-level improvement with resmetirom compared with placebo (42.2% vs. 31.1%, *p*=0.01), and at the stricter 10% threshold for MCID, additional benefits were seen in Abdominal Symptoms and Role Physical (*p*≤0.05) (Table 3).

**TABLE 2:** Changes in HRQL scores in resmetirom-treated patients with early MASH with MRI-PDFF response versus placebo

MRI-PDFF response (30%)	Response with resmetirom (100 mg or 80 mg)	Placebo
Abdominal symptoms	0.24 (0.12–0.37)^a^ ^b^	−0.01 (−0.18 to 0.17)
Worry	0.22 (0.12–0.32)^a^	0.08 (−0.07 to 0.23)
Total CLDQ-NAFLD score	0.09 (0.01–0.17)^a^	0.01 (−0.11 to 0.13)
Role Physical	0.51 (−1.71 to 2.74)	−2.74 (−4.93 to −0.56)^a^
Vitality	0.81 (−1.00 to 2.62)	−1.77 (−3.75 to 0.20)^a^
Role Emotional	−0.56 (−2.67 to 1.55)	−2.81 (−5.02 to −0.60)^a^
Mental Component Summary	−0.18 (−1.01 to 0.65)	−0.89 (−1.86 to 0.09)^a^
Effects of liver disease	−1.94 (−3.31 to −0.58)^a^	−1.90 (−3.65 to −0.15)^a^
Concentration/memory	−1.84 (−3.50 to −0.17)^a^	−3.23 (−5.35 to −1.12)^a^
Health distress	1.94 (0.20–3.68)^a^	0.49 (−2.00 to 2.98)
Sleep	−1.73 (−3.48 to 0.02)^a^	−0.92 (−3.04 to 1.20)
Stigma of liver disease	2.18 (0.44–3.91)^a^	0.60 (−1.93 to 3.14)
MRI-PDFF response (50%)		
Abdominal symptoms	0.23 (0.08–0.39)^a^ ^b^	−0.01 (−0.18 to 0.17)
Worry	0.19 (0.07–0.31)^a^	0.08 (−0.07 to 0.23)
Total CLDQ-NAFLD score	0.07 (−0.02 to 0.16)^a^	0.01 (−0.11 to 0.13)
Role Physical	0.68 (−1.86 to 3.21)^b^	−2.74 (−4.93 to −0.56)^a^
Vitality	2.01 (−0.12 to 4.13)^b^	−1.77 (−3.75-0.20)^a^
Role Emotional	−1.87 (−4.11 to 0.37)	−2.81 (−5.02 to −0.60)^a^
Physical Component Summary	0.59 (−0.22 to 1.41)^a^	0.01 (−0.77 to 0.80)
Mental Component Summary	−0.41 (−1.33 to 0.50)	−0.89 (−1.86 to 0.09)^a^
Effects of liver disease	−2.32 (−3.80 to −0.84)^a^	−1.90 (−3.65 to −0.15)^a^
Concentration/memory	−2.70 (−4.73 to −0.67)^a^	−3.23 (−5.35 to −1.12)^a^
Sleep	−2.00 (−4.07 to 0.08)^a^	−0.92 (−3.04 to 1.20)
Stigma of liver disease	2.35 (0.18–4.52)^a^	0.60 (−1.93 to 3.14)

Each cell shows the mean change from baseline to week 52 with 95% CI.

^a^ Two-sided *p*<0.05 versus zero change (within-treatment comparison, indicates significant change from baseline).

^b^ Two-sided *p*<0.05 versus placebo. Only statistically significant changes (two-sided *p*<0.05 in comparison to zero or to placebo) are included; all HRQL domains are shown in Supplemental Table S7, http://links.lww.com/HC9/C262.

Abbreviations: CLDQ-NAFLD, Chronic Liver Disease Questionnaire–nonalcoholic fatty liver disease; HRQL, health-related quality of life; MASH, metabolic dysfunction–associated steatohepatitis; MRI-PDFF, magnetic resonance imaging–proton density fat fraction.

**TABLE 3:** Proportions of patients with early MASH with MRI-PDFF response (≥30% reduction from baseline) meeting MCID for improvement in select HRQL scores (Table 2) versus placebo

	Response with resmetirom (100 mg or 80 mg)	Placebo	*p*
MCID = 5% of the score range size			
Abdominal symptoms	44.4%	38.5%	0.17
Worry	32.5%	25.7%	0.08
Total CLDQ-NAFLD score	34.0%	30.7%	0.42
Role Physical (RP)	33.0%	27.6%	0.17
Vitality (VT)	42.2%	31.1%	0.0072
Role Emotional (RE)	22.1%	17.5%	0.18
Effects of liver disease	20.1%	20.6%	0.88
Concentration/memory	29.6%	25.9%	0.33
Health distress	21.8%	20.2%	0.63
Sleep	33.9%	36.4%	0.54
Stigma of liver disease	36.2%	32.9%	0.41
Sensitivity analysis: MCID = 3%			
Abdominal symptoms	44.4%	38.5%	0.17
Worry	36.1%	30.7%	0.19
Total CLDQ-NAFLD score	43.8%	37.6%	0.15
Role Physical (RP)	33.3%	27.6%	0.15
Vitality (VT)	42.2%	31.1%	0.0072
Role Emotional (RE)	22.1%	17.5%	0.18
Effects of liver disease	20.1%	20.6%	0.88
Concentration/memory	29.6%	25.9%	0.33
Health distress	21.8%	20.2%	0.63
Sleep	37.1%	39.5%	0.56
Stigma of liver disease	36.2%	32.9%	0.41
Sensitivity analysis: MCID = 10%			
Abdominal symptoms	32.0%	24.3%	0.0509
Worry	25.1%	18.8%	0.08
Total CLDQ-NAFLD score	17.2%	16.1%	0.73
Role Physical (RP)	24.4%	16.2%	0.0185
Vitality (VT)	26.4%	19.3%	0.0485
Role Emotional (RE)	16.7%	11.0%	0.06
Effects of liver disease	10.9%	11.0%	0.99
Concentration/memory	17.0%	15.4%	0.61
Health distress	21.8%	20.2%	0.63
Sleep	22.4%	24.6%	0.55
Stigma of liver disease	22.7%	23.7%	0.78

Abbreviations: HRQL, health-related quality of life; MASH, metabolic dysfunction–associated steatohepatitis; MICD, minimal clinically important difference; MRI-PDFF, magnetic resonance imaging–proton density fat fraction; CLDQ-NAFLD, Chronic Liver Disease Questionnaire–nonalcoholic fatty liver disease.

### HRQL in well-compensated MASH cirrhosis

There were 180 patients with compensated cirrhosis included in the study. At baseline, HRQL scores of patients with MASH cirrhosis were significantly lower (up to −15% of score range) for all 6 domains of CLDQ-NAFLD and 13/17 domains of LDQoL in comparison to patients with early MASH, and for 2/6 domains and the total score of CLDQ-NAFLD, 5/17 domains and 2/3 total scores of LDQoL in comparison to those with F2–F3 (*p*<0.05) (Table 4). Among patients with MASH cirrhosis, there was no significant difference in baseline HRQL scores between those with MRI-PDFF ≤5% and >5% (all *p*>0.05) (Supplemental Table S8, http://links.lww.com/HC9/C262).

**TABLE 4:** Baseline HRQL scores in subjects with MASLD/MASH by fibrosis severity

HRQL score	Cirrhosis	MASH with F2–F3^a^	Early MASH	*p*-value cirrhosis vs. early MASH	*p*-value cirrhosis vs. F2–F3 MASH
N	180	966	1143		
CLDQ-NAFLD					
Abdominal symptoms	5.21±1.54	5.41±1.49	5.50±1.44	0.0216	0.11
Activity/energy	5.18±1.46	5.45±1.34	5.58±1.29	0.0015	0.0405
Emotional function	5.36±1.14	5.43±1.22	5.56±1.13	0.0190	0.24
Fatigue	4.56±1.47	4.72±1.43	4.90±1.37	0.0083	0.22
Systemic symptoms	4.95±1.29	5.16±1.23	5.31±1.17	0.0011	0.06
Worry	5.17±1.45	5.64±1.37	6.08±1.18	<0.0001	0.0001
Total score	5.07±1.16	5.30±1.12	5.49±1.05	<0.0001	0.0189
LDQoL					
Physical Functioning (PF)	68.5±27.9	74.3±24.9	77.1±23.0	0.0004	0.0248
Role Physical (RP)	69.7±27.4	75.5±26.6	78.8±24.2	<0.0001	0.0082
Bodily Pain (BP)	63.5±25.2	65.9±25.1	69.2±23.2	0.0079	0.27
General Health (GH)	56.6±20.6	58.8±19.8	64.8±19.4	<0.0001	0.15
Vitality (VT)	53.4±22.2	56.5±21.7	60.1±20.0	0.0004	0.09
Social Functioning (SF)	77.9±23.9	81.8±23.8	85.1±20.9	0.0001	0.0253
Role Emotional (RE)	82.7±22.9	83.2±23.2	86.9±20.1	0.0248	0.69
Mental Health (MH)	76.1±17.1	74.9±18.4	77.8±17.0	0.20	0.52
Physical Component Summary (PCS)	44.7±10.6	47.1±9.5	48.5±8.8	<0.0001	0.0130
Mental Component Summary (MCS)	51.3±9.5	51.0±10.1	52.5±8.8	0.10	0.90
Symptoms of liver disease	76.0±18.8	77.2±17.9	80.6±16.6	0.0026	0.50
Effects of liver disease	76.1±15.3	77.7±14.7	81.4±13.7	<0.0001	0.12
Concentration/memory	78.5±20.8	79.7±21.4	82.9±19.1	0.0067	0.32
Health distress	76.6±24.1	81.5±24.0	89.3±19.3	<0.0001	0.0051
Sexual Function	78.1±21.7	79.7±24.9	83.2±21.5	0.0170	0.19
Sleep	61.8±20.4	63.0±20.0	64.9±19.2	0.05	0.38
Loneliness	83.3±19.1	83.3±19.7	85.1±18.5	0.16	0.87
Hopelessness	74.4±23.7	78.8±21.4	83.2±19.1	<0.0001	0.0358
Stigma of liver disease	84.1±18.1	83.9±19.3	85.1±18.6	0.29	0.77
Total score	76.2±13.3	78.1±13.9	81.5±12.1	<0.0001	0.0333

^a^ Historic MAESTRO-NASH sample.^13^

Abbreviations: HRQL, health-related quality of life; LDQoL, Liver Disease Quality of Life; MASH, metabolic dysfunction–associated steatohepatitis; MASLD, metabolic dysfunction–associated steatotic liver disease; CLDQ-NAFLD, Chronic Liver Disease Questionnaire–nonalcoholic fatty liver disease.

**FIGURE 2:**
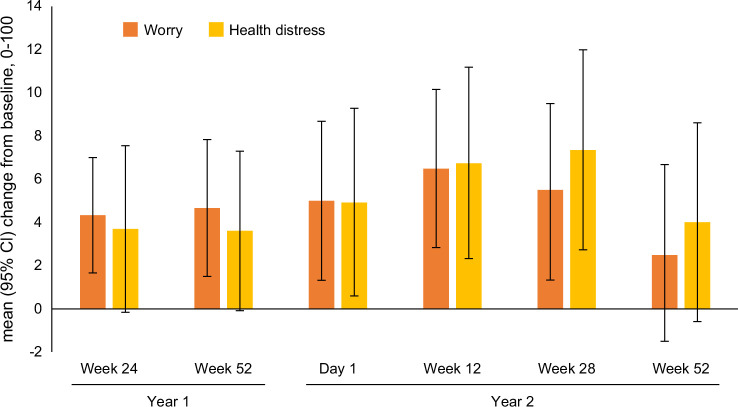
Changes in select HRQL scores in resmetirom-treated patients with MASH cirrhosis (mean change from baseline with 95% CI; *p* value for comparison vs. zero). All HRQL scores are shown in Supplemental Table S9, http://links.lww.com/HC9/C262. Abbreviations: HRQL, health-related quality of life; MASH, metabolic dysfunction–associated steatohepatitis.

In patients with MASH cirrhosis, by week 24 of treatment with resmetirom, there were improvements in Worry (CLDQ-NAFLD) and Health Distress (LDQOL) up to +4% of score range which sustained by week 52 and throughout year 2 of treatment (day 1, week 12, week 28) (*p*<0.05) (Figure 2 and Supplemental Table S9, http://links.lww.com/HC9/C262). In MASH cirrhotic subjects with MRI-PDFF >5%, PDFF response by week 52, there was an improvement in Stigma score (LDQoL): mean change (+3.9 vs. −4.2, *p*=0.042) (Supplemental Table S10, http://links.lww.com/HC9/C262). In contrast, nonresponder patients experienced significant worsening of Loneliness and Hopelessness scores (up to −8%), which was not observed in MRI-PDFF responders with cirrhosis (*p*>0.05) (Supplemental Table S10, http://links.lww.com/HC9/C262). Treatment response assessed at year 2 week 52 was found to be associated with superior scores in the Effect of liver disease (mean change from baseline +0.90 vs. −6.40) and Loneliness (+4.6 vs. −4.4) domains of LDQoL (*p*<0.05) (Supplemental Table S10, http://links.lww.com/HC9/C262). At the same time, there was statistically significant worsening in Concentration/memory scores of LDQoL among treatment responders at year 2 week 52, which was not observed in nonresponders (mean change −4.45 vs. +2.25) (Supplemental Table S10, http://links.lww.com/HC9/C262).

There was a significant improvement in Health Distress scores seen at year 2 week 52 in patients with MASH cirrhosis and VCTE response: mean change from year 1 baseline +9.9 versus −1.8 in nonresponders (*p*<0.05) (Supplemental Table S11, http://links.lww.com/HC9/C262).

## DISCUSSION

In this large cohort of MASLD patients, including both early MASH and MASH cirrhosis cases, enrolled in the MAESTRO-NAFLD program, we demonstrate that treatment with resmetirom is associated with clinically meaningful improvements across multiple domains of HRQL. These improvements were particularly notable among patients who achieved a reduction in hepatic steatosis, as measured by MRI-PDFF. These findings add a novel, patient-centered dimension to the clinical profile of resmetirom, which has previously been shown to improve histologic features of steatohepatitis and fibrosis in MASH.^12^,^13^ Several key observations emerge from these data.

Patients with MASLD commonly experience reductions in HRQL. While these impairments are typically mild in non-cirrhotic individuals, they become significantly more pronounced with advancing liver disease.^17^,^18^,^19^,^20^,^21^ In this study, MRI-PDFF was significantly correlated with worsening HRQL scores in domains such as General Health, Vitality, Mental Health, and Stigma among patients with early MASH. Moreover, comparisons between patients with early MASH and those with MASH cirrhosis revealed that individuals with cirrhosis reported substantially lower HRQL across nearly all domains at baseline. These findings are consistent with prior studies showing that HRQL deteriorates in parallel with increasing liver disease severity.^17^,^18^,^19^,^20^,^21^

Among patients with early MASH, resmetirom generally attenuated the declines in physical function and fatigue observed in the placebo group over the 52-week treatment period. The positive effects in comparison to patients’ own baseline levels or to placebo were notable in the domains of Role Physical, Vitality, and Health Distress, and were accompanied by improvements in liver-specific symptoms such as Abdominal Symptoms and Worry. Notably, the HRQL benefits were most pronounced in patients who achieved ≥30% or ≥50% relative reductions in MRI-PDFF, supporting the notion that improvements in this key surrogate marker of liver health may translate into tangible gains in patient well-being. This finding aligns with previous studies demonstrating that greater reductions in liver fat by MRI-PDFF are associated with improvements in metabolic and inflammatory parameters, suggesting a potential mechanistic link between hepatic fat reduction and symptom relief in MASLD.^13^,^22^ Furthermore, several domains showed clinically meaningful improvement, with up to 45% of resmetirom-treated patients exceeding the MCID threshold, although these effects varied by domain.

In patients with well-compensated MASH cirrhosis, treatment with resmetirom was also associated with sustained improvements in emotional well-being, particularly in the domains of Worry and Health Distress. Among MRI-PDFF responders, additional benefits included a reversal of negative trends in social isolation and perceived stigma. These improvements are especially noteworthy given the limited therapeutic options currently available for patients with MASH-related cirrhosis. Interestingly, some HRQL domain scores related to Sleep, Concentration/memory, and Symptoms of liver disease showed declines (worsening) over time. In patients with cirrhosis, the decline in cognitive function may reflect neuropsychiatric status with disease progression, and general worsening of symptoms may also reflect the natural history of this advanced disease. The underlying mechanisms and clinical consequences of the observed declines should be investigated with long-term outcomes assessments.

When interpreting the findings of the MAESTRO-NAFLD-1 study, it is important to consider the patient population in the context of other resmetirom clinical trial cohorts.^13^ Notably, the early MASH arm of MAESTRO-NAFLD-1 primarily included patients with early fibrosis (F1), defined by liver stiffness measurements ranging from ≥5.5 to <8.5 kPa. In contrast, the previously published MAESTRO-NASH trial enrolled patients with biopsy-confirmed MASH and more advanced fibrosis (predominantly F2–F3),^12^,^13^ while the cirrhotic arm of MAESTRO-NAFLD-1 comprised individuals with well-compensated F4 disease. Together, these cohorts represent a continuum of disease severity ranging from early MASH to fibrotic MASH and ultimately to MASH cirrhosis. In the context of treatment with resmetirom, the extent of HRQL improvement observed in each patient group may reflect both the baseline symptom burden in patients with different disease severity, but also the potential for clinical benefit. For example, cirrhotic patients exhibited notable improvements in domains such as Worry, Health Distress, and Stigma, while non-cirrhotic patients experienced more pronounced gains in Abdominal Symptoms and Worry. These patterns underscore the importance of tailoring therapeutic expectations and endpoints based on the disease stage.

Our findings should be interpreted in light of several limitations. First, although the large sample size and prospective data collection enhanced the internal validity of the study, the patient population was limited to clinical trial participants who met specific inclusion and exclusion criteria. As such, the generalizability of these results to broader, more diverse real-world populations may be limited; future studies in less selected cohorts are warranted to confirm these findings in routine clinical settings. The study was also not prospectively powered to detect differences in HRQL outcomes, particularly in patients with MASH cirrhosis. Furthermore, although no significant differences were observed between open-label and double-blind resmetirom groups receiving the same dose, the possibility of reporting bias related to open-label treatment cannot be fully excluded. In addition, while MRI-PDFF is a well-validated and sensitive marker of hepatic steatosis, the relationship between HRQL improvements and other clinically meaningful outcomes, such as histologic fibrosis or noninvasive tests of fibrosis, remains to be elucidated. Further research is also needed to determine whether changes in HRQL correlate with long-term liver-related outcomes.

In summary, treatment with resmetirom was associated with meaningful improvements in some domains of HRQL across the full spectrum of MASLD severity, with the greatest benefits observed among patients who achieved a reduction in hepatic fat as measured by MRI-PDFF. These improvements were evident in both early MASH and MASH cirrhosis populations, highlighting the potential of resmetirom to alleviate the symptomatic burden of disease. As the therapeutic landscape for MASLD continues to advance, the incorporation of HRQL measures as key endpoints in clinical trials is essential to ensure that emerging treatments deliver tangible benefits in patients’ daily functioning and overall well-being.

## Supplementary Material

SUPPLEMENTARY MATERIAL
