# Supplementary material for: Improvement in health-related quality of life after treatment with resmetirom in patients with the spectrum of MASLD: From early MASH to MASH cirrhosis
Source: Hepatol Commun. 2026 Feb 26;10(3):e00913. doi: 10.1097/HC9.0000000000000913 (PMC12947990; doi:10.1097/HC9.0000000000000913)

**Appendix for:**

**Improvement in Health-Related Quality of Life After Treatment with Resmetirom in Patients with the Spectrum of MASLD: From Early MASH to MASH Cirrhosis**

Zobair M. Younossi ^1,2^, Fatema Nader ^1,2^, Dominic Labriola ^3^, Rebecca Taub ^3^, Andrei Racila ^1,2,4^, Linda Henry ^1,2,4^, Maria Stepanova ^1,2,4^

1. The Global NASH/MASH Council, Washington, DC, United States,
2. Beatty Liver and Obesity Research Program, Inova Health System, Falls Church, VA, United States
3. Madrigal Pharmaceuticals, West Conshohocken, PA, United States,
4. Center for Outcomes Research in Liver Diseases, Washington, DC, United States

**Supplementary Table 1.** Comparison of baseline HRQL scores of early MASH patients by the treatment arm.

| HRQL score | Double-blind | | | Open-label resmetirom 100 mg | All early MASH |
| --- | --- | --- | --- | --- | --- |
|  | Resmetirom 100 mg | Resmetirom 80 mg | placebo |  |  |
| N | 325 | 327 | 320 | 171 | 1143 |
| Abdominal symptoms | 5.61 ± 1.39 | 5.43 ± 1.44 | 5.56 ± 1.44 | 5.33 ± 1.57 | 5.50 ± 1.44 |
| Activity/energy | 5.63 ± 1.33 | 5.60 ± 1.31 | 5.57 ± 1.20 | 5.46 ± 1.33 | 5.58 ± 1.29 |
| Emotional function | 5.62 ± 1.12 | 5.49 ± 1.14 | 5.59 ± 1.12 | 5.51 ± 1.14 | 5.56 ± 1.13 |
| Fatigue | 4.93 ± 1.40 | 4.83 ± 1.37 | 5.02 ± 1.26 | 4.75 ± 1.51 | 4.90 ± 1.37 |
| Systemic symptoms | 5.40 ± 1.20 | 5.21 ± 1.19 | 5.39 ± 1.06 | 5.15 ± 1.23 | 5.31 ± 1.17 |
| Worry | 6.11 ± 1.18 | 6.12 ± 1.13 | 6.07 ± 1.19 | 5.97 ± 1.28 | 6.08 ± 1.18 |
| Total CLDQ-NAFLD | 5.55 ± 1.05 | 5.45 ± 1.07 | 5.53 ± 0.99 | 5.36 ± 1.10 | 5.49 ± 1.05 |
| Physical Functioning (PF) | 79.0 ± 21.9 | 75.5 ± 24.2 | 78.2 ± 22.1 | 74.4 ± 23.7 | 77.1 ± 23.0 |
| Role Physical (RP) | 80.1 ± 24.0 | 77.7 ± 24.4 | 79.8 ± 23.2 | 76.1 ± 26.1 | 78.8 ± 24.2 |
| Bodily Pain (BP) | 71.3 ± 22.7 | 67.6 ± 23.8 | 70.8 ± 22.7 | 65.4 ± 23.3 | 69.2 ± 23.2 |
| General Health (GH) | 65.7 ± 19.1 | 63.9 ± 20.0 | 64.5 ± 19.0 | 65.7 ± 19.7 | 64.8 ± 19.4 |
| Vitality (VT) | 61.1 ± 19.4 | 59.0 ± 19.9 | 61.2 ± 19.6 | 57.9 ± 21.7 | 60.1 ± 20.0 |
| Social Functioning (SF) | 86.4 ± 20.5 | 84.9 ± 20.9 | 85.2 ± 21.1 | 82.8 ± 21.6 | 85.1 ± 20.9 |
| Role Emotional (RE) | 88.1 ± 19.8 | 86.6 ± 19.6 | 86.9 ± 20.2 | 85.0 ± 21.6 | 86.9 ± 20.1 |
| Mental Health (MH) | 78.5 ± 16.8 | 76.9 ± 17.0 | 77.8 ± 17.5 | 78.2 ± 16.6 | 77.8 ± 17.0 |
| Physical Summary (PCS) | 49.2 ± 8.4 | 47.9 ± 9.3 | 49.0 ± 8.4 | 47.4 ± 9.2 | 48.5 ± 8.8 |
| Mental Summary (MCS) | 52.9 ± 8.6 | 52.4 ± 8.7 | 52.5 ± 9.2 | 52.3 ± 8.9 | 52.5 ± 8.8 |
| Symptoms of liver disease | 81.6 ± 16.8 | 79.6 ± 17.1 | 81.9 ± 15.9 | 78.3 ± 16.8 | 80.6 ± 16.6 |
| Effects of liver disease | 81.5 ± 14.2 | 81.8 ± 13.8 | 81.6 ± 13.1 | 80.0 ± 13.9 | 81.4 ± 13.7 |
| Concentration/memory | 83.3 ± 19.1 | 81.6 ± 19.6 | 84.4 ± 17.5 | 81.7 ± 20.6 | 82.9 ± 19.1 |
| Health distress | 89.4 ± 20.3 | 89.7 ± 18.7 | 89.1 ± 18.8 | 88.6 ± 19.6 | 89.3 ± 19.3 |
| Sexual Function | 83.9 ± 20.8 | 83.6 ± 20.1 | 83.5 ± 23.1 | 80.4 ± 22.4 | 83.2 ± 21.5 |
| Sleep | 65.5 ± 19.3 | 63.6 ± 20.0 | 66.1 ± 18.4 | 64.3 ± 18.9 | 64.9 ± 19.2 |
| Loneliness | 85.8 ± 18.1 | 83.8 ± 18.8 | 85.2 ± 18.6 | 86.3 ± 18.5 | 85.1 ± 18.5 |
| Hopelessness | 84.2 ± 17.7 | 82.1 ± 18.7 | 82.8 ± 20.6 | 84.1 ± 19.6 | 83.2 ± 19.1 |
| Stigma of liver disease | 86.9 ± 17.8 | 85.6 ± 17.3 | 84.6 ± 18.4 | 81.8 ± 22.2 | 85.1 ± 18.6 |
| Total LDQOL | 82.2 ± 11.9 | 81.0 ± 12.0 | 81.9 ± 12.4 | 80.5 ± 12.1 | 81.5 ± 12.1 |

**Supplementary Table 2**. Correlations of baseline HRQL scores with baseline MRI-PDFF (Spearman’s) in patients with early MASH.

| HRQL score | Correlation | P |
| --- | --- | --- |
| Abdominal symptoms | -0.06 | 0.08 |
| Activity/energy | 0.00 | 0.95 |
| Emotional function | -0.04 | 0.18 |
| Fatigue | -0.05 | 0.10 |
| Systemic symptoms | 0.00 | 0.94 |
| Worry | -0.06 | 0.06 |
| Total CLDQ-NAFLD score | -0.04 | 0.22 |
| Physical Functioning (PF) | -0.01 | 0.84 |
| Role Physical (RP) | -0.03 | 0.33 |
| Bodily Pain (BP) | -0.01 | 0.74 |
| General Health (GH) | -0.06 | 0.0478 |
| Vitality (VT) | -0.10 | 0.0008 |
| Social Functioning (SF) | -0.02 | 0.49 |
| Role Emotional (RE) | -0.04 | 0.15 |
| Mental Health (MH) | -0.04 | 0.22 |
| Physical Component Summary (PCS) | -0.03 | 0.40 |
| Mental Component Summary (MCS) | -0.06 | 0.0405 |
| Symptoms of liver disease | -0.04 | 0.18 |
| Effects of liver disease | -0.05 | 0.12 |
| Concentration/memory | 0.02 | 0.61 |
| Health distress | -0.01 | 0.63 |
| Sexual Function | 0.02 | 0.71 |
| Sleep | -0.05 | 0.15 |
| Loneliness | -0.01 | 0.82 |
| Hopelessness | -0.02 | 0.50 |
| Stigma of liver disease | -0.07 | 0.0177 |
| Total LDQOL | -0.04 | 0.20 |

**Supplementary Table 3**. The changes in HRQL scores in 100 mg double-blind and 100 mg open-label resmetirom groups of patients with early MASH (LSM with 97.5% CI returned by MMRM, p0 vs. zero [p vs. open-label]).

| HRQL score | Resmetirom 100 mg double-blind | Resmetirom 100 mg open-label |
| --- | --- | --- |
| Week 24 | | |
| Abdominal symptoms | 0.23 (-0.01-0.47), p0=0.0319 [p=0.92] | 0.22 (-0.07-0.50), p0=0.08 |
| Activity/energy | -0.04 (-0.25-0.17), p0=0.69 [p=0.96] | -0.04 (-0.29-0.20), p0=0.70 |
| Emotional function | -0.04 (-0.23-0.14), p0=0.60 [p=0.17] | 0.09 (-0.13-0.31), p0=0.38 |
| Fatigue | -0.07 (-0.29-0.16), p0=0.50 [p=0.77] | -0.04 (-0.30-0.23), p0=0.75 |
| Systemic symptoms | -0.00 (-0.19-0.19), p0=1.00 [p=0.66] | 0.04 (-0.18-0.26), p0=0.68 |
| Worry | 0.16 (-0.02-0.34), p0=0.0451 [p=0.07] | 0.32 (0.11-0.53), p0=0.0008 |
| Total CLDQ-NAFLD score | 0.04 (-0.12-0.20), p0=0.58 [p=0.37] | 0.11 (-0.08-0.30), p0=0.20 |
| Physical Functioning (PF) | -2.32 (-5.93-1.29), p0=0.15 [p=0.16] | -4.78 (-9.03- -0.52), p0=0.0120 |
| Role Physical (RP) | -1.06 (-5.10-2.98), p0=0.55 [p=0.30] | -3.13 (-7.88-1.63), p0=0.14 |
| Bodily Pain (BP) | -1.77 (-5.88-2.33), p0=0.33 [p=0.31] | -3.80 (-8.64-1.03), p0=0.08 |
| General Health (GH) | -0.58 (-3.57-2.42), p0=0.67 [p=0.75] | -1.06 (-4.58-2.47), p0=0.50 |
| Vitality (VT) | -0.39 (-3.92-3.14), p0=0.81 [p=0.94] | -0.26 (-4.41-3.90), p0=0.89 |
| Social Functioning (SF) | -0.04 (-4.05-3.96), p0=0.98 [p=0.45] | 1.44 (-3.26-6.15), p0=0.49 |
| Role Emotional (RE) | -2.21 (-6.19-1.76), p0=0.21 [p=0.94] | -2.07 (-6.74-2.59), p0=0.32 |
| Mental Health (MH) | -2.07 (-5.12-0.98), p0=0.13 [p=0.37] | -0.71 (-4.30-2.88), p0=0.65 |
| Physical Summary (PCS) | -0.40 (-1.75-0.96), p0=0.51 [p=0.09] | -1.50 (-3.10-0.10), p0=0.0350 |
| Mental summary (MCS) | -0.65 (-2.31-1.02), p0=0.38 [p=0.19] | 0.42 (-1.54-2.37), p0=0.63 |
| Symptoms of liver disease | -1.58 (-4.54-1.39), p0=0.23 [p=0.31] | -3.05 (-6.54-0.44), p0=0.05 |
| Effects of liver disease | -0.96 (-3.18-1.27), p0=0.33 [p=0.99] | -0.97 (-3.59-1.64), p0=0.40 |
| Concentration/memory | 0.46 (-2.69-3.61), p0=0.74 [p=0.49] | 1.53 (-2.17-5.22), p0=0.35 |
| Health distress | 1.65 (-1.53-4.83), p0=0.24 [p=0.63] | 2.40 (-1.34-6.14), p0=0.15 |
| Sexual Function | 3.42 (-7.36-14.19), p0=0.47 [p=0.74] | 2.34 (-8.67-13.35), p0=0.63 |
| Sleep | -1.99 (-5.27-1.30), p0=0.17 [p=0.65] | -1.25 (-5.11-2.61), p0=0.47 |
| Loneliness | -3.20 (-6.51-0.11), p0=0.0304 [p=0.79] | -2.75 (-6.65-1.15), p0=0.11 |
| Hopelessness | -1.17 (-4.54-2.21), p0=0.44 [p=0.15] | 1.31 (-2.67-5.30), p0=0.46 |
| Stigma of liver disease | -0.34 (-3.48-2.80), p0=0.81 [p=0.05] | 2.85 (-0.89-6.59), p0=0.09 |
| Total LDQOL | -0.72 (-2.67-1.22), p0=0.40 [p=0.32] | 0.25 (-2.05-2.55), p0=0.81 |
| Week 52 | | |
| Abdominal symptoms | 0.33 (0.10-0.56), p0=0.0016 [p=0.67] | 0.38 (0.11-0.65), p0=0.0019 |
| Activity/energy | -0.08 (-0.30-0.14), p0=0.40 [p=0.71] | -0.12 (-0.38-0.14), p0=0.29 |
| Emotional function | 0.06 (-0.13-0.24), p0=0.48 [p=0.87] | 0.04 (-0.17-0.26), p0=0.65 |
| Fatigue | -0.03 (-0.26-0.20), p0=0.75 [p=0.62] | -0.09 (-0.36-0.18), p0=0.46 |
| Systemic symptoms | -0.01 (-0.20-0.18), p0=0.88 [p=0.31] | -0.11 (-0.33-0.11), p0=0.27 |
| Worry | 0.18 (-0.00-0.37), p0=0.0272 [p=0.68] | 0.22 (0.00-0.44), p0=0.0235 |
| Total CLDQ-NAFLD score | 0.08 (-0.09-0.24), p0=0.29 [p=0.87] | 0.06 (-0.13-0.25), p0=0.46 |
| Physical Functioning (PF) | -2.02 (-5.60-1.57), p0=0.21 [p=0.42] | -3.44 (-7.64-0.77), p0=0.07 |
| Role Physical (RP) | -0.54 (-4.53-3.45), p0=0.76 [p=0.73] | -1.22 (-5.89-3.44), p0=0.56 |
| Bodily Pain (BP) | -1.13 (-5.29-3.02), p0=0.54 [p=0.17] | -4.03 (-8.95-0.88), p0=0.07 |
| General Health (GH) | -0.58 (-3.53-2.37), p0=0.66 [p=0.88] | -0.36 (-3.81-3.08), p0=0.81 |
| Vitality (VT) | -0.74 (-4.25-2.77), p0=0.64 [p=1.00] | -0.73 (-4.84-3.38), p0=0.69 |
| Social Functioning (SF) | 0.79 (-3.26-4.83), p0=0.66 [p=0.94] | 0.93 (-3.82-5.68), p0=0.66 |
| Role Emotional (RE) | 0.06 (-3.89-4.02), p0=0.97 [p=0.41] | -1.53 (-6.16-3.09), p0=0.46 |
| Mental Health (MH) | -0.77 (-3.77-2.24), p0=0.57 [p=0.72] | -0.25 (-3.75-3.26), p0=0.87 |
| Physical Summary (PCS) | -0.53 (-1.89-0.83), p0=0.38 [p=0.41] | -1.08 (-2.68-0.52), p0=0.13 |
| Mental summary (MCS) | 0.05 (-1.61-1.71), p0=0.95 [p=0.74] | 0.32 (-1.62-2.26), p0=0.71 |
| Symptoms of liver disease | -1.75 (-4.72-1.21), p0=0.18 [p=0.20] | -3.65 (-7.13- -0.16), p0=0.0189 |
| Effects of liver disease | -0.61 (-2.89-1.68), p0=0.55 [p=0.43] | -1.54 (-4.24-1.16), p0=0.20 |
| Concentration/memory | 0.99 (-2.19-4.18), p0=0.48 [p=0.39] | -0.35 (-4.09-3.38), p0=0.83 |
| Health distress | 3.18 (0.01-6.36), p0=0.0247 [p=0.40] | 1.87 (-1.85-5.59), p0=0.26 |
| Sexual Function | 1.96 (-8.74-12.67), p0=0.68 [p=0.32] | 5.29 (-5.77-16.35), p0=0.28 |
| Sleep | -2.52 (-5.83-0.80), p0=0.09 [p=0.85] | -2.84 (-6.74-1.05), p0=0.10 |
| Loneliness | -0.56 (-3.75-2.63), p0=0.69 [p=0.04] | -3.75 (-7.46- -0.05), p0=0.0232 |
| Hopelessness | -1.01 (-4.27-2.26), p0=0.49 [p=0.09] | 1.74 (-2.07-5.56), p0=0.31 |
| Stigma of liver disease | 1.79 (-1.22-4.79), p0=0.18 [p=0.40] | 0.54 (-2.98-4.06), p0=0.73 |
| Total LDQOL | -0.01 (-1.88-1.87), p0=1.00 [p=0.48] | -0.63 (-2.83-1.56), p0=0.52 |

**Supplementary Table 4**. The changes in HRQL scores of patients with early MASH by treatment regimen (LSM w/97.5% CI by MMRM; * p<0.05 vs. zero; & p<0.05 vs. placebo).

| HRQL score | Resmetirom 100 mg (pooled) | Resmetirom 80 mg | placebo |
| --- | --- | --- | --- |
| Week 24 | | | |
| Abdominal symptoms | 0.15 (-0.02-0.31) * | 0.14 (-0.04-0.33) | 0.10 (-0.09-0.28) |
| Activity/energy | -0.03 (-0.17-0.12) | -0.04 (-0.20-0.13) | -0.10 (-0.26-0.07) |
| Emotional function | -0.02 (-0.15-0.11) | 0.03 (-0.12-0.18) | -0.03 (-0.17-0.12) |
| Fatigue | -0.04 (-0.19-0.12) | -0.04 (-0.22-0.13) | -0.03 (-0.21-0.14) |
| Systemic symptoms | 0.01 (-0.12-0.15) | -0.04 (-0.19-0.11) | -0.06 (-0.21-0.09) |
| Worry | 0.17 (0.04-0.30) * & | 0.16 (0.01-0.30) * | 0.04 (-0.11-0.18) |
| Total CLDQ-NAFLD score | 0.04 (-0.07-0.16) | 0.03 (-0.09-0.16) | -0.02 (-0.14-0.11) |
| Physical Functioning (PF) | -2.71 (-5.19- -0.22) * | -2.22 (-5.07-0.62) | -3.45 (-6.26- -0.63) * |
| Role Physical (RP) | -1.20 (-4.06-1.65) & | -2.35 (-5.61-0.90) | -5.50 (-8.72- -2.27) * |
| Bodily Pain (BP) | -2.65 (-5.46-0.17) * | -2.33 (-5.55-0.88) | -3.51 (-6.69- -0.33) * |
| General Health (GH) | -0.32 (-2.43-1.80) | -0.92 (-3.32-1.49) | -0.67 (-3.06-1.71) |
| Vitality (VT) | -0.56 (-2.94-1.82) | -1.73 (-4.44-0.98) | -2.70 (-5.39- -0.01) * |
| Social Functioning (SF) | -0.20 (-2.97-2.57) | -0.48 (-3.65-2.69) | -2.22 (-5.36-0.92) |
| Role Emotional (RE) | -2.48 (-5.18-0.23) * | -1.35 (-4.44-1.74) | -3.51 (-6.58- -0.45) * |
| Mental Health (MH) | -1.49 (-3.60-0.61) | -0.68 (-3.09-1.72) | -1.76 (-4.15-0.63) |
| Physical Summary (PCS) | -0.58 (-1.53-0.36) | -0.84 (-1.91-0.24) | -1.36 (-2.43- -0.29) * |
| Mental summary (MCS) | -0.51 (-1.64-0.62) | -0.27 (-1.55-1.02) | -0.87 (-2.15-0.41) |
| Symptoms of liver disease | -1.17 (-3.18-0.84) | -1.30 (-3.59-0.98) | -1.72 (-3.98-0.54) |
| Effects of liver disease | -1.43 (-3.11-0.26) | -0.89 (-2.83-1.06) | -1.76 (-3.68-0.16) * |
| Concentration/memory | -0.30 (-2.51-1.91) & | 0.00 (-2.51-2.51) & | -2.72 (-5.21- -0.23) * |
| Health distress | 1.33 (-0.98-3.63) & | 2.36 (-0.28-4.99) * & | -1.25 (-3.86-1.36) |
| Sexual Function | 1.37 (-3.80-6.54) | -1.26 (-6.74-4.21) | -2.60 (-7.94-2.74) |
| Sleep | -1.22 (-3.43-1.00) | 0.46 (-2.06-2.98) | -0.37 (-2.86-2.13) |
| Loneliness | -1.64 (-3.93-0.65) | -2.56 (-5.18-0.06) * | -2.14 (-4.73-0.45) |
| Hopelessness | 0.04 (-2.37-2.46) | 0.90 (-1.87-3.66) | -1.12 (-3.85-1.62) |
| Stigma of liver disease | 0.77 (-1.38-2.91) | 1.52 (-0.95-3.99) | 0.17 (-2.27-2.61) |
| Total LDQOL | -0.32 (-1.68-1.04) | -0.11 (-1.65-1.44) | -1.41 (-2.94-0.12) * |
| Week 52 | | | |
| **Abdominal symptoms** | 0.28 (0.11-0.44) * & | 0.28 (0.09-0.48) * & | 0.05 (-0.14-0.24) |
| Activity/energy | -0.09 (-0.24-0.06) | -0.09 (-0.26-0.09) | 0.01 (-0.17-0.18) |
| Emotional function | 0.03 (-0.11-0.16) | 0.04 (-0.12-0.19) | -0.03 (-0.18-0.12) |
| Fatigue | -0.04 (-0.20-0.13) | 0.01 (-0.18-0.20) | -0.11 (-0.30-0.08) |
| Systemic symptoms | -0.05 (-0.19-0.09) | 0.01 (-0.15-0.17) | -0.06 (-0.22-0.10) |
| **Worry** | 0.15 (0.02-0.28) * | 0.22 (0.07-0.38) * & | 0.04 (-0.11-0.20) |
| Total CLDQ-NAFLD score | 0.05 (-0.07-0.17) | 0.08 (-0.06-0.22) | -0.02 (-0.16-0.11) |
| Physical Functioning (PF) | -2.07 (-4.53-0.39) | -3.07 (-5.89- -0.24) * | -1.41 (-4.21-1.39) |
| Role Physical (RP) | -0.19 (-3.04-2.66) & | -3.17 (-6.44-0.10) * | -3.59 (-6.84- -0.34) * |
| Bodily Pain (BP) | -2.42 (-5.30-0.46) | -2.25 (-5.59-1.08) | -2.25 (-5.55-1.05) |
| General Health (GH) | -0.08 (-2.26-2.10) | 0.12 (-2.39-2.62) | 0.09 (-2.40-2.58) |
| Vitality (VT) | -0.97 (-3.38-1.43) | -0.79 (-3.55-1.98) | -1.49 (-4.23-1.26) |
| Social Functioning (SF) | 0.10 (-2.64-2.84) | -0.07 (-3.22-3.09) | -0.38 (-3.50-2.75) |
| Role Emotional (RE) | -0.86 (-3.54-1.82) | -1.14 (-4.21-1.93) | -3.00 (-6.05-0.04) * |
| Mental Health (MH) | -0.53 (-2.61-1.55) | 1.56 (-0.82-3.95) & | -0.93 (-3.30-1.44) |
| Physical Summary (PCS) | -0.54 (-1.50-0.43) | -1.21 (-2.32- -0.10) * | -0.65 (-1.75-0.45) |
| Mental summary (MCS) | -0.12 (-1.25-1.01) | 0.58 (-0.72-1.88) | -0.69 (-1.98-0.60) |
| Symptoms of liver disease | -1.52 (-3.61-0.56) | -1.90 (-4.31-0.50) | -1.35 (-3.73-1.04) |
| Effects of liver disease | -1.39 (-3.06-0.29) | -1.76 (-3.70-0.18) * | -1.51 (-3.44-0.41) |
| Concentration/memory | -0.66 (-2.95-1.62) | -1.52 (-4.15-1.11) | -2.28 (-4.90-0.33) |
| **Health distress** | 2.07 (-0.21-4.36) * | 3.25 (0.62-5.87) * & | 0.09 (-2.51-2.69) |
| Sexual Function | 1.40 (-3.84-6.65) | -0.02 (-5.74-5.70) | -3.22 (-8.68-2.23) |
| Sleep | -2.16 (-4.46-0.13) * | -1.15 (-3.80-1.50) | -1.69 (-4.32-0.93) |
| Loneliness | -0.37 (-2.67-1.92) | -1.75 (-4.40-0.90) | -1.47 (-4.10-1.16) |
| Hopelessness | 0.25 (-2.19-2.69) | 0.68 (-2.15-3.51) | -0.73 (-3.53-2.07) |
| Stigma of liver disease | 1.29 (-0.93-3.52) | 1.71 (-0.90-4.32) | 0.51 (-2.06-3.09) |
| Total LDQOL | -0.21 (-1.61-1.20) | -0.35 (-1.97-1.27) | -1.29 (-2.90-0.31) |

**Supplementary Table 5**. Proportions of patients with early MASH meeting the MCID for improvement in each HRQL score (p-value vs. placebo returned by a logistic regression).

| HRQL score | Resmetirom 100 mg | Resmetirom 80 mg | placebo |
| --- | --- | --- | --- |
| Week 24 | | | |
| Abdominal symptoms | 39.4% p=0.60 | 40.9% p=0.65 | 36.3% |
| Activity/energy | 33.0% p=0.98 | 29.1% p=0.39 | 32.1% |
| Emotional function | 32.7% p=0.16 | 35.7% p=0.06 | 26.5% |
| Fatigue | 38.8% p=0.98 | 32.6% p=0.14 | 35.9% |
| Systemic symptoms | 36.2% p=0.27 | 32.2% p=0.82 | 30.8% |
| Worry | 28.7% p=0.0469 | 23.9% p=0.17 | 22.2% |
| Total CLDQ-NAFLD score | 30.1% p=0.15 | 25.7% p=0.92 | 23.5% |
| Physical Functioning (PF) | 36.8% p=0.50 | 38.9% p=0.36 | 32.7% |
| Role Physical (RP) | 33.2% p=0.0101 | 31.8% p=0.06 | 22.2% |
| Bodily Pain (BP) | 30.4% p=0.41 | 32.2% p=0.41 | 25.4% |
| General Health (GH) | 36.1% p=0.98 | 32.2% p=0.15 | 36.4% |
| Vitality (VT) | 39.1% p=0.28 | 37.7% p=0.56 | 33.1% |
| Social Functioning (SF) | 23.3% p=0.32 | 20.1% p=0.97 | 19.4% |
| Role Emotional (RE) | 21.0% p=0.0236 | 18.4% p=0.07 | 14.2% |
| Mental Health (MH) | 33.2% p=0.90 | 31.8% p=0.73 | 32.3% |
| Symptoms of liver disease | 30.9% p=0.31 | 26.8% p=0.64 | 25.8% |
| Effects of liver disease | 24.3% p=0.32 | 25.1% p=0.10 | 19.8% |
| Concentration/memory | 29.2% p=0.87 | 32.6% p=0.31 | 25.4% |
| Health distress | 21.7% p=0.0015 | 18.4% p=0.0050 | 14.9% |
| Sexual Function | 34.0% p=0.0017 | 29.8% p=0.0097 | 17.0% |
| Sleep | 38.1% p=0.65 | 42.7% p=0.65 | 37.9% |
| Loneliness | 29.9% p=0.13 | 31.4% p=0.15 | 25.4% |
| Hopelessness | 32.8% p=0.0047 | 31.8% p=0.0317 | 24.2% |
| Stigma of liver disease | 33.5% p=0.0170 | 31.0% p=0.19 | 27.0% |
| Total LDQOL | 22.5% p=0.0266 | 22.6% p=0.0368 | 15.3% |
| Week 52 | | | |
| Abdominal symptoms | 42.6% p=0.63 | 45.0% p=0.48 | 38.5% |
| Activity/energy | 32.4% p=0.0415 | 28.2% p=0.0061 | 38.5% |
| Emotional function | 34.9% p=0.42 | 37.8% p=0.15 | 30.3% |
| Fatigue | 36.4% p=0.27 | 36.4% p=0.31 | 38.1% |
| Systemic symptoms | 33.5% p=0.17 | 39.2% p=0.87 | 37.2% |
| Worry | 30.4% p=0.37 | 30.6% p=0.06 | 25.7% |
| Total CLDQ-NAFLD score | 31.0% p=0.60 | 34.0% p=0.79 | 30.7% |
| Physical Functioning (PF) | 35.9% p=0.56 | 36.7% p=0.49 | 36.8% |
| Role Physical (RP) | 33.1% p=0.47 | 29.8% p=0.83 | 27.6% |
| Bodily Pain (BP) | 32.6% p=0.50 | 29.4% p=0.71 | 28.1% |
| General Health (GH) | 35.9% p=0.76 | 39.0% p=1.00 | 38.2% |
| Vitality (VT) | 39.5% p=0.13 | 39.4% p=0.19 | 31.1% |
| Social Functioning (SF) | 24.3% p=0.12 | 21.6% p=0.49 | 18.0% |
| Role Emotional (RE) | 22.1% p=0.22 | 21.6% p=0.12 | 17.5% |
| Mental Health (MH) | 36.5% p=0.39 | 45.4% p=0.0064 | 32.9% |
| Symptoms of liver disease | 32.0% p=0.51 | 28.4% p=0.08 | 32.0% |
| Effects of liver disease | 21.5% p=0.74 | 20.6% p=0.99 | 20.6% |
| Concentration/memory | 30.7% p=0.67 | 27.5% p=0.69 | 25.9% |
| Health distress | 23.8% p=0.19 | 17.4% p=0.88 | 20.2% |
| Sexual Function | 33.3% p=0.0064 | 36.5% p=0.0060 | 20.0% |
| Sleep | 34.5% p=0.47 | 36.2% p=0.61 | 36.4% |
| Loneliness | 31.8% p=0.15 | 33.0% p=0.24 | 27.2% |
| Hopelessness | 29.8% p=0.77 | 32.1% p=0.67 | 29.4% |
| Stigma of liver disease | 33.4% p=0.99 | 32.1% p=0.79 | 32.9% |
| Total LDQOL | 22.4% p=0.57 | 22.5% p=0.53 | 19.3% |

**Supplementary Table 6**. Changes in HRQL scores in resmetirom-treated patients with early MASH (100 mg and 80 mg separately) with MRI-PDFF response (≥30% or ≥50% cutoff) vs. placebo (full arm). Each cell shows mean change from baseline to Week 52 with 95% CI; * p<0.05 vs. zero (within-treatment comparison, indicates significant change from baseline); & p<0.05 vs. comparator (placebo). The 100 mg and 80 mg groups pooled are shown in **Supplementary Table 7**.

| HRQL score | Response with resmetirom 100 mg | Response with resmetirom 80 mg | | Placebo |
| --- | --- | --- | --- | --- |
| **MRI-PDFF response (30%)** | | | | |
| Abdominal symptoms | 0.18 (0.02-0.33) * | 0.38 (0.17-0.59) * & | | -0.01 (-0.18-0.17) |
| Activity/energy | 0.02 (-0.12-0.16) | 0.06 (-0.11-0.22) | | 0.09 (-0.05-0.23) * |
| Emotional function | 0.02 (-0.09-0.13) | 0.06 (-0.09-0.22) | | -0.02 (-0.14-0.11) |
| Fatigue | 0.03 (-0.13-0.18) | 0.06 (-0.13-0.25) | | -0.05 (-0.20-0.10) |
| Systemic symptoms | -0.09 (-0.20-0.03) | 0.13 (-0.05-0.30) | | -0.05 (-0.17-0.08) |
| Worry | 0.21 (0.09-0.34) * | 0.24 (0.08-0.39) * | | 0.08 (-0.07-0.23) |
| Total CLDQ-NAFLD score | 0.06 (-0.04-0.16) | 0.15 (0.02-0.29) * | | 0.01 (-0.11-0.13) |
| Physical Functioning (PF) | 0.54 (-1.76-2.83) | 0.51 (-2.37-3.39) | | 0.59 (-1.30-2.47) |
| Role Physical (RP) | 1.05 (-1.72-3.81) & | -0.53 (-4.26-3.19) | | -2.74 (-4.93- -0.56) * |
| Bodily Pain (BP) | -0.77 (-3.40-1.85) | 0.62 (-2.33-3.58) | | -1.08 (-3.64-1.48) |
| General Health (GH) | 0.48 (-1.44-2.40) | 0.79 (-1.97-3.56) | | 0.92 (-1.22-3.06) |
| Vitality (VT) | 0.68 (-1.61-2.96) | 1.07 (-1.87-4.01) | | -1.77 (-3.75-0.20) * |
| Social Functioning (SF) | 0.27 (-2.58-3.12) | -0.96 (-3.95-2.03) | | -0.82 (-3.27-1.63) |
| Role Emotional (RE) | -0.45 (-3.18-2.27) | -0.78 (-4.03-2.46) | | -2.81 (-5.02- -0.60) * |
| Mental Health (MH) | -1.00 (-2.78-0.79) | 0.90 (-1.24-3.04) | | -0.76 (-2.61-1.08) |
| Physical Component Summary (PCS) | 0.34 (-0.56-1.23) | 0.17 (-0.98-1.31) | | 0.01 (-0.77-0.80) |
| Mental Component summary (MCS) | -0.30 (-1.39-0.80) | 0.05 (-1.12-1.23) | | -0.89 (-1.86-0.09) * |
| Symptoms of liver disease | -1.67 (-3.50-0.17) | -0.28 (-2.77-2.20) | | -0.80 (-2.73-1.14) |
| Effects of liver disease | -1.37 (-3.03-0.29) | -3.08 (-5.47- -0.68) * | | -1.90 (-3.65- -0.15) * |
| Concentration/memory | -1.58 (-3.77-0.61) | -2.35 (-4.76-0.06) | | -3.23 (-5.35- -1.12) * |
| Health distress | 2.22 (-0.16-4.60) | 1.39 (-0.81-3.59) | | 0.49 (-2.00-2.98) |
| Sexual Function | -2.78 (-6.79-1.22) | -0.08 (-6.19-6.03) | | -3.84 (-7.41- -0.27) |
| Sleep | -2.36 (-4.60- -0.13) * | -0.47 (-3.24-2.30) | | -0.92 (-3.04-1.20) |
| Loneliness | 0.40 (-1.58-2.37) | -0.90 (-3.99-2.19) | | -1.20 (-3.17-0.78) |
| Hopelessness | 0.22 (-1.99-2.43) | 1.35 (-1.97-4.68) | | -0.40 (-3.00-2.19) |
| Stigma of liver disease | 2.17 (0.04-4.31) * | 2.19 (-0.81-5.19) | | 0.60 (-1.93-3.14) |
| Total LDQOL | -0.23 (-1.38-0.92) | -0.36 (-2.01-1.29) | | -1.13 (-2.56-0.30) |
| **MRI-PDFF response (50%)** | | | | |
| Abdominal symptoms | 0.18 (-0.01-0.37) | | 0.36 (0.08-0.63) * & | -0.01 (-0.18-0.17) |
| Activity/energy | 0.02 (-0.14-0.19) | | 0.00 (-0.19-0.19) | 0.09 (-0.05-0.23) * |
| Emotional function | -0.02 (-0.14-0.10) | | -0.01 (-0.19-0.17) | -0.02 (-0.14-0.11) |
| Fatigue | 0.02 (-0.16-0.20) | | 0.05 (-0.18-0.29) | -0.05 (-0.20-0.10) |
| Systemic symptoms | -0.08 (-0.21-0.06) | | 0.07 (-0.14-0.28) | -0.05 (-0.17-0.08) |
| Worry | 0.20 (0.05-0.35) * | | 0.16 (-0.03-0.36) | 0.08 (-0.07-0.23) |
| Total CLDQ-NAFLD score | 0.05 (-0.06-0.17) | | 0.11 (-0.06-0.27) | 0.01 (-0.11-0.13) |
| Physical Functioning (PF) | 1.23 (-1.16-3.61) | | 0.45 (-3.21-4.11) | 0.59 (-1.30-2.47) |
| Role Physical (RP) | 1.86 (-1.16-4.88) & | | -1.87 (-6.51-2.77) | -2.74 (-4.93- -0.56) * |
| Bodily Pain (BP) | 1.07 (-1.88-4.02) | | -0.70 (-4.46-3.06) | -1.08 (-3.64-1.48) |
| General Health (GH) | 0.96 (-1.19-3.10) | | -0.56 (-4.03-2.91) | 0.92 (-1.22-3.06) |
| Vitality (VT) | 2.75 (0.12-5.38) * & | | 0.41 (-3.18-3.99) | -1.77 (-3.75-0.20) * |
| Social Functioning (SF) | 0.23 (-2.82-3.27) | | -2.11 (-5.48-1.26) | -0.82 (-3.27-1.63) |
| Role Emotional (RE) | -1.03 (-3.99-1.94) | | -3.68 (-6.71- -0.65) * | -2.81 (-5.02- -0.60) * |
| Mental Health (MH) | -0.60 (-2.59-1.38) | | 0.00 (-2.44-2.44) | -0.76 (-2.61-1.08) |
| Physical Component Summary (PCS) | 0.87 (-0.12-1.87) * | | -0.01 (-1.41-1.39) | 0.01 (-0.77-0.80) |
| Mental Component summary (MCS) | -0.25 (-1.48-0.98) | | -0.77 (-1.96-0.41) | -0.89 (-1.86-0.09) * |
| Symptoms of liver disease | -1.06 (-3.32-1.21) | | -0.39 (-3.44-2.66) | -0.80 (-2.73-1.14) |
| Effects of liver disease | -2.11 (-3.95- -0.27) * | | -2.77 (-5.25- -0.29) * | -1.90 (-3.65- -0.15) * |
| Concentration/memory | -2.26 (-4.89-0.37) | | -3.65 (-6.67- -0.64) * | -3.23 (-5.35- -1.12) * |
| Health distress | 1.43 (-1.30-4.16) | | 0.65 (-2.28-3.58) | 0.49 (-2.00-2.98) |
| Sexual Function | -2.82 (-7.38-1.73) | | 0.86 (-7.00-8.73) | -3.84 (-7.41- -0.27) |
| Sleep | -2.68 (-5.27- -0.09) * | | -0.52 (-3.93-2.89) | -0.92 (-3.04-1.20) |
| Loneliness | 1.45 (-0.80-3.71) | | -1.10 (-4.59-2.38) | -1.20 (-3.17-0.78) |
| Hopelessness | 0.20 (-2.59-3.00) | | 0.11 (-4.30-4.52) | -0.40 (-3.00-2.19) |
| Stigma of liver disease | 3.21 (0.66-5.77) * | | 0.49 (-3.56-4.54) | 0.60 (-1.93-3.14) |
| Total LDQOL | -0.22 (-1.58-1.14) | | -0.99 (-3.10-1.12) | -1.13 (-2.56-0.30) |

**Supplementary Table 7**. Changes in HRQL scores in resmetirom-treated patients with early MASH (100 mg and 80 mg pooled) with MRI-PDFF response (≥30% or ≥50% cutoff) vs. placebo (full arm). Each cell shows mean change from baseline to Week 52 with 95% CI; * p<0.05 vs. zero (within-treatment comparison, indicates significant change from baseline); & p<0.05 vs. comparator (placebo). The 100 mg and 80 mg groups separately are shown in **Supplementary Table 6**.

| **MRI-PDFF response (30%)** | Response with resmetirom | Placebo |
| --- | --- | --- |
| Abdominal symptoms | 0.24 (0.12-0.37) * & | -0.01 (-0.18-0.17) |
| Activity/energy | 0.03 (-0.08-0.14) | 0.09 (-0.05-0.23) * |
| Emotional function | 0.03 (-0.06-0.13) | -0.02 (-0.14-0.11) |
| Fatigue | 0.04 (-0.09-0.16) | -0.05 (-0.20-0.10) |
| Systemic symptoms | -0.02 (-0.11-0.08) | -0.05 (-0.17-0.08) |
| Worry | 0.22 (0.12-0.32) * | 0.08 (-0.07-0.23) |
| Total CLDQ-NAFLD score | 0.09 (0.01-0.17) * | 0.01 (-0.11-0.13) |
| Physical Functioning (PF) | 0.53 (-1.28-2.33) | 0.59 (-1.30-2.47) |
| Role Physical (RP) | 0.51 (-1.71-2.74) | -2.74 (-4.93- -0.56) * |
| Bodily Pain (BP) | -0.30 (-2.31-1.70) | -1.08 (-3.64-1.48) |
| General Health (GH) | 0.58 (-0.99-2.16) | 0.92 (-1.22-3.06) |
| Vitality (VT) | 0.81 (-1.00-2.62) | -1.77 (-3.75-0.20) * |
| Social Functioning (SF) | -0.14 (-2.28-2.00) | -0.82 (-3.27-1.63) |
| Role Emotional (RE) | -0.56 (-2.67-1.55) | -2.81 (-5.02- -0.60) * |
| Mental Health (MH) | -0.36 (-1.75-1.03) | -0.76 (-2.61-1.08) |
| Physical Component Summary (PCS) | 0.28 (-0.43-0.98) | 0.01 (-0.77-0.80) |
| Mental Component summary (MCS) | -0.18 (-1.01-0.65) | -0.89 (-1.86-0.09) * |
| Symptoms of liver disease | -1.20 (-2.68-0.27) | -0.80 (-2.73-1.14) |
| Effects of liver disease | -1.94 (-3.31- -0.58) * | -1.90 (-3.65- -0.15) * |
| Concentration/memory | -1.84 (-3.50- -0.17) * | -3.23 (-5.35- -1.12) * |
| Health distress | 1.94 (0.20-3.68) * | 0.49 (-2.00-2.98) |
| Sexual Function | -1.88 (-5.24-1.47) | -3.84 (-7.41- -0.27) |
| Sleep | -1.73 (-3.48-0.02) * | -0.92 (-3.04-1.20) |
| Loneliness | -0.04 (-1.71-1.63) | -1.20 (-3.17-0.78) |
| Hopelessness | 0.60 (-1.24-2.44) | -0.40 (-3.00-2.19) |
| Stigma of liver disease | 2.18 (0.44-3.91) * | 0.60 (-1.93-3.14) |
| Total LDQOL | -0.27 (-1.21-0.67) | -1.13 (-2.56-0.30) |
| **MRI-PDFF response (50%)** | Response with resmetirom | Placebo |
| **Abdominal symptoms** | 0.23 (0.08-0.39) * & | -0.01 (-0.18-0.17) |
| Activity/energy | 0.01 (-0.11-0.14) | 0.09 (-0.05-0.23) * |
| Emotional function | -0.02 (-0.12-0.08) | -0.02 (-0.14-0.11) |
| Fatigue | 0.03 (-0.12-0.17) | -0.05 (-0.20-0.10) |
| Systemic symptoms | -0.03 (-0.14-0.08) | -0.05 (-0.17-0.08) |
| **Worry** | 0.19 (0.07-0.31) * | 0.08 (-0.07-0.23) |
| Total CLDQ-NAFLD score | 0.07 (-0.02-0.16) * | 0.01 (-0.11-0.13) |
| Physical Functioning (PF) | 0.98 (-1.01-2.98) | 0.59 (-1.30-2.47) |
| Role Physical (RP) | 0.68 (-1.86-3.21) & | -2.74 (-4.93- -0.56) * |
| Bodily Pain (BP) | 0.51 (-1.83-2.85) | -1.08 (-3.64-1.48) |
| General Health (GH) | 0.48 (-1.35-2.31) | 0.92 (-1.22-3.06) |
| Vitality (VT) | 2.01 (-0.12-4.13) & | -1.77 (-3.75-0.20) * |
| Social Functioning (SF) | -0.51 (-2.85-1.82) | -0.82 (-3.27-1.63) |
| Role Emotional (RE) | -1.87 (-4.11-0.37) | -2.81 (-5.02- -0.60) * |
| Mental Health (MH) | -0.41 (-1.97-1.15) | -0.76 (-2.61-1.08) |
| Physical Component Summary (PCS) | 0.59 (-0.22-1.41) * | 0.01 (-0.77-0.80) |
| Mental Component summary (MCS) | -0.41 (-1.33-0.50) | -0.89 (-1.86-0.09) * |
| Symptoms of liver disease | -0.84 (-2.67-0.98) | -0.80 (-2.73-1.14) |
| Effects of liver disease | -2.32 (-3.80- -0.84) * | -1.90 (-3.65- -0.15) * |
| Concentration/memory | -2.70 (-4.73- -0.67) * | -3.23 (-5.35- -1.12) * |
| Health distress | 1.18 (-0.90-3.27) | 0.49 (-2.00-2.98) |
| Sexual Function | -1.67 (-5.64-2.31) | -3.84 (-7.41- -0.27) |
| Sleep | -2.00 (-4.07-0.08) * | -0.92 (-3.04-1.20) |
| Loneliness | 0.64 (-1.25-2.54) | -1.20 (-3.17-0.78) |
| Hopelessness | 0.17 (-2.19-2.53) | -0.40 (-3.00-2.19) |
| Stigma of liver disease | 2.35 (0.18-4.52) * | 0.60 (-1.93-3.14) |
| Total LDQOL | -0.46 (-1.60-0.68) | -1.13 (-2.56-0.30) |

**Supplementary Table 8**. Baseline HRQL scores of patients with MASH cirrhosis by baseline PDFF.

| HRQL score | baseline PDFF >5% | baseline PDFF <=5% | p |
| --- | --- | --- | --- |
| N | 126 | 42 |  |
| Abdominal symptoms | 5.28 ± 1.54 | 5.20 ± 1.58 | 0.78 |
| Activity/energy | 5.25 ± 1.45 | 5.08 ± 1.53 | 0.56 |
| Emotional function | 5.35 ± 1.17 | 5.37 ± 1.10 | 0.98 |
| Fatigue | 4.53 ± 1.48 | 4.59 ± 1.48 | 0.80 |
| Systemic symptoms | 4.99 ± 1.33 | 4.89 ± 1.25 | 0.63 |
| Worry | 5.15 ± 1.45 | 5.20 ± 1.58 | 0.84 |
| Total CLDQ-NASH score | 5.09 ± 1.16 | 5.05 ± 1.24 | 0.97 |
| Physical Functioning (PF) | 69.2 ± 28.0 | 67.4 ± 27.8 | 0.62 |
| Role Physical (RP) | 70.8 ± 27.3 | 68.9 ± 28.1 | 0.58 |
| Bodily Pain (BP) | 64.5 ± 25.9 | 61.1 ± 23.6 | 0.53 |
| General Health (GH) | 56.1 ± 20.7 | 57.8 ± 19.7 | 0.73 |
| Vitality (VT) | 52.7 ± 22.2 | 55.4 ± 22.8 | 0.40 |
| Social Functioning (SF) | 77.4 ± 24.3 | 78.4 ± 23.3 | 0.85 |
| Role Emotional (RE) | 82.0 ± 24.3 | 85.4 ± 19.5 | 0.56 |
| Mental Health (MH) | 76.3 ± 17.0 | 74.1 ± 17.3 | 0.38 |
| Physical Component Summary (PCS) | 45.1 ± 10.7 | 44.3 ± 10.7 | 0.60 |
| Mental Component Summary (MCS) | 50.9 ± 9.6 | 51.7 ± 9.1 | 0.72 |
| Symptoms of liver disease | 77.3 ± 18.8 | 73.0 ± 17.9 | 0.12 |
| Effects of liver disease | 75.7 ± 15.6 | 75.3 ± 15.7 | 0.65 |
| Concentration/memory | 78.0 ± 21.3 | 78.2 ± 21.1 | 0.89 |
| Health distress | 75.4 ± 25.4 | 78.7 ± 22.0 | 0.58 |
| Sexual Function | 76.3 ± 22.9 | 87.0 ± 12.6 | 0.21 |
| Sleep | 61.2 ± 20.8 | 62.4 ± 19.2 | 0.67 |
| Loneliness | 83.6 ± 18.7 | 83.0 ± 20.0 | 0.77 |
| Hopelessness | 76.9 ± 21.8 | 68.0 ± 27.4 | 0.09 |
| Stigma of liver disease | 84.2 ± 17.1 | 83.1 ± 22.2 | 0.64 |
| Total LDQOL | 76.4 ± 13.5 | 75.3 ± 13.8 | 0.71 |

**Supplementary Table 9**. Changes in HRQL scores in resmetirom-treated subjects with MASH cirrhosis (mean change from baseline with 95% CI; p value for comparison vs. zero).

| HRQL score / Year 1 | Change from baseline to week 24 | p | Change from baseline to week 52 | p |
| --- | --- | --- | --- | --- |
| Abdominal symptoms | 0.08 (-0.15-0.31) | 0.44 | -0.00 (-0.21-0.21) | 0.77 |
| Activity/energy | 0.05 (-0.11-0.20) | 0.98 | -0.10 (-0.27-0.08) | 0.21 |
| Emotional function | 0.04 (-0.09-0.17) | 0.75 | 0.04 (-0.11-0.19) | 0.54 |
| Fatigue | 0.01 (-0.14-0.17) | 0.75 | -0.05 (-0.23-0.12) | 0.48 |
| Systemic symptoms | -0.03 (-0.17-0.11) | 0.67 | -0.10 (-0.23-0.03) | 0.18 |
| **Worry** | 0.26 (0.10-0.42) | 0.0003 | 0.28 (0.09-0.47) | 0.0012 |
| Total CLDQ-NASH score | 0.07 (-0.05-0.18) | 0.44 | 0.01 (-0.11-0.14) | 0.75 |
| Physical Functioning (PF) | -0.88 (-3.52-1.77) | 0.72 | 0.58 (-2.88-4.03) | 0.62 |
| Role Physical (RP) | -0.29 (-3.26-2.69) | 0.79 | 1.45 (-2.05-4.95) | 0.64 |
| Bodily Pain (BP) | -0.77 (-3.69-2.15) | 0.65 | -1.49 (-4.83-1.85) | 0.39 |
| General Health (GH) | -1.50 (-3.69-0.69) | 0.11 | -1.23 (-3.52-1.05) | 0.25 |
| Vitality (VT) | -1.54 (-4.01-0.94) | 0.37 | -0.57 (-3.28-2.13) | 0.81 |
| Social Functioning (SF) | 1.48 (-1.80-4.76) | 0.49 | 1.06 (-2.41-4.52) | 0.62 |
| Role Emotional (RE) | -0.44 (-3.11-2.24) | 0.64 | 2.35 (-1.25-5.94) | 0.11 |
| Mental Health (MH) | -1.30 (-3.51-0.91) | 0.12 | -0.46 (-2.86-1.94) | 0.37 |
| Physical Component Summary (PCS) | -0.30 (-1.34-0.75) | 0.48 | -0.21 (-1.39-0.98) | 0.98 |
| Mental Component Summary (MCS) | -0.29 (-1.47-0.89) | 0.95 | 0.31 (-1.02-1.64) | 0.71 |
| Symptoms of liver disease | -1.64 (-4.04-0.75) | 0.09 | -0.89 (-3.05-1.27) | 0.65 |
| Effects of liver disease | -0.61 (-2.82-1.59) | 0.23 | -2.54 (-4.90- -0.17) | 0.0182 |
| Concentration/memory | -2.67 (-5.11- -0.23) | 0.0269 | -2.07 (-4.63-0.50) | 0.11 |
| **Health distress** | 3.70 (-0.15-7.55) | 0.0429 | 3.61 (-0.08-7.30) | 0.0354 |
| Sexual Function | 2.56 (-2.80-7.92) | 0.34 | 0.21 (-7.44-7.86) | 0.77 |
| Sleep | -1.37 (-3.37-0.62) | 0.08 | -1.30 (-3.74-1.13) | 0.43 |
| Loneliness | 1.40 (-1.23-4.03) | 0.17 | -0.39 (-3.35-2.57) | 0.24 |
| Hopelessness | 0.05 (-2.97-3.08) | 0.77 | -0.29 (-3.76-3.17) | 0.64 |
| Stigma of liver disease | -0.12 (-2.41-2.17) | 0.98 | 1.19 (-1.60-3.97) | 0.53 |
| Total LDQOL | -0.04 (-1.47-1.39) | 0.90 | -0.36 (-1.89-1.17) | 0.70 |
| Year 2: day 1 and week 12 | Change from baseline to Year 2 day 1 | p | Change from baseline to Year 2 week 12 | p |
| Abdominal symptoms | 0.11 (-0.12-0.35) | 0.39 | 0.05 (-0.23-0.32) | 0.67 |
| Activity/energy | -0.15 (-0.34-0.05) | 0.10 | -0.11 (-0.30-0.09) | 0.29 |
| Emotional function | 0.03 (-0.14-0.20) | 0.55 | 0.07 (-0.09-0.23) | 0.56 |
| Fatigue | -0.06 (-0.27-0.15) | 0.67 | -0.06 (-0.27-0.15) | 0.55 |
| Systemic symptoms | -0.12 (-0.27-0.04) | 0.08 | -0.21 (-0.37- -0.04) | 0.0119 |
| Worry | 0.30 (0.08-0.52) | 0.0086 | 0.39 (0.17-0.61) | 0.0003 |
| Total CLDQ-NASH score | 0.02 (-0.13-0.17) | 0.63 | 0.02 (-0.13-0.17) | 0.46 |
| Physical Functioning (PF) | 0.15 (-3.29-3.58) | 0.82 | -2.06 (-5.54-1.41) | 0.39 |
| Role Physical (RP) | 1.15 (-2.68-4.98) | 0.78 | -1.55 (-5.34-2.24) | 0.49 |
| Bodily Pain (BP) | 1.71 (-2.32-5.74) | 0.35 | -0.93 (-5.04-3.19) | 0.73 |
| General Health (GH) | -1.86 (-4.54-0.82) | 0.28 | -3.46 (-6.21- -0.71) | 0.0235 |
| Vitality (VT) | -1.32 (-4.53-1.89) | 0.52 | -1.27 (-4.52-1.98) | 0.33 |
| Social Functioning (SF) | 0.11 (-3.56-3.78) | 0.88 | 1.66 (-2.04-5.36) | 0.36 |
| Role Emotional (RE) | 0.07 (-3.73-3.88) | 0.90 | -0.15 (-3.41-3.11) | 0.85 |
| Mental Health (MH) | 0.26 (-2.68-3.21) | 0.80 | -0.62 (-3.40-2.17) | 0.33 |
| Physical Component Summary (PCS) | 0.11 (-1.25-1.48) | 0.81 | -1.00 (-2.37-0.37) | 0.19 |
| Mental Component Summary (MCS) | -0.17 (-1.81-1.46) | 0.78 | 0.16 (-1.26-1.59) | 0.52 |
| Symptoms of liver disease | -1.14 (-3.69-1.41) | 0.38 | -2.04 (-4.83-0.76) | 0.07 |
| Effects of liver disease | -0.88 (-3.45-1.69) | 0.35 | -0.47 (-3.18-2.24) | 0.34 |
| Concentration/memory | 0.71 (-2.40-3.82) | 0.59 | -0.39 (-2.96-2.19) | 0.79 |
| Health distress | 4.93 (0.60-9.27) | 0.0369 | 6.75 (2.32-11.18) | 0.0035 |
| Sexual Function | -4.19 (-13.75-5.37) | 0.59 | -1.63 (-9.23-5.98) | 1.00 |
| Sleep | 0.88 (-2.40-4.15) | 0.58 | 0.04 (-2.60-2.69) | 0.98 |
| Loneliness | -1.23 (-3.76-1.31) | 0.16 | -0.18 (-3.04-2.69) | 0.86 |
| Hopelessness | -1.54 (-5.25-2.18) | 0.28 | -1.03 (-5.12-3.06) | 0.63 |
| Stigma of liver disease | -0.38 (-3.22-2.46) | 0.49 | -0.72 (-4.12-2.69) | 0.78 |
| Total LDQOL | 0.12 (-1.77-2.01) | 0.74 | 0.23 (-1.58-2.03) | 0.54 |
| Year 2: week 28 and week 52 | Change from baseline to Year 2 week 28 | p | Change from baseline to Year 2 week 52 | p |
| Abdominal symptoms | -0.06 (-0.35-0.24) | 0.49 | 0.07 (-0.20-0.34) | 0.70 |
| Activity/energy | -0.21 (-0.43-0.01) | 0.0114 | -0.16 (-0.38-0.06) | 0.08 |
| Emotional function | -0.02 (-0.19-0.16) | 0.44 | -0.01 (-0.18-0.17) | 0.69 |
| Fatigue | -0.17 (-0.39-0.05) | 0.09 | -0.10 (-0.32-0.13) | 0.51 |
| Systemic symptoms | -0.21 (-0.38- -0.05) | 0.0063 | -0.14 (-0.31-0.03) | 0.22 |
| **Worry** | 0.33 (0.08-0.57) | 0.0050 | 0.15 (-0.09-0.40) | 0.14 |
| Total CLDQ-NASH score | -0.06 (-0.22-0.10) | 0.33 | -0.03 (-0.18-0.12) | 0.88 |
| Physical Functioning (PF) | -2.74 (-6.01-0.53) | 0.0327 | -3.24 (-6.49-0.01) | 0.0270 |
| Role Physical (RP) | -2.22 (-5.75-1.31) | 0.10 | -1.00 (-4.71-2.71) | 0.70 |
| Bodily Pain (BP) | -4.29 (-8.39- -0.19) | 0.05 | -1.38 (-5.54-2.79) | 0.48 |
| General Health (GH) | -4.50 (-7.69- -1.32) | 0.0016 | -3.77 (-6.80- -0.74) | 0.0294 |
| Vitality (VT) | -3.56 (-6.72- -0.41) | 0.0355 | -2.71 (-5.92-0.49) | 0.13 |
| Social Functioning (SF) | -0.12 (-4.46-4.23) | 0.97 | -1.53 (-5.68-2.62) | 0.45 |
| Role Emotional (RE) | -0.23 (-3.35-2.88) | 0.98 | -1.34 (-5.12-2.44) | 0.56 |
| Mental Health (MH) | -0.28 (-3.10-2.54) | 0.46 | -1.89 (-4.93-1.16) | 0.10 |
| Physical Component Summary (PCS) | -1.81 (-3.25- -0.36) | 0.0088 | -0.98 (-2.28-0.31) | 0.29 |
| Mental Component Summary (MCS) | 0.02 (-1.38-1.42) | 0.78 | -0.78 (-2.29-0.74) | 0.47 |
| Symptoms of liver disease | -2.37 (-5.28-0.54) | 0.0186 | -2.26 (-5.13-0.60) | 0.15 |
| Effects of liver disease | -1.68 (-4.59-1.22) | 0.12 | -2.01 (-4.88-0.85) | 0.18 |
| Concentration/memory | -1.05 (-4.21-2.10) | 0.29 | -2.18 (-5.12-0.76) | 0.06 |
| **Health distress** | 7.36 (2.74-11.98) | 0.0019 | 4.01 (-0.59-8.61) | 0.09 |
| Sexual Function | -5.17 (-15.08-4.74) | 0.08 | -5.14 (-14.42-4.14) | 0.50 |
| Sleep | -0.28 (-3.39-2.83) | 0.54 | -0.47 (-3.47-2.53) | 0.56 |
| Loneliness | -1.37 (-4.24-1.51) | 0.48 | 2.03 (-0.66-4.72) | 0.06 |
| Hopelessness | -0.86 (-4.80-3.09) | 0.89 | -3.46 (-7.04-0.12) | 0.06 |
| Stigma of liver disease | 2.16 (-0.82-5.14) | 0.06 | -0.83 (-4.41-2.76) | 0.99 |
| Total LDQOL | 0.28 (-1.64-2.21) | 0.94 | -0.74 (-2.40-0.92) | 0.25 |

**Supplementary Table 10**. Changes in HRQL scores in resmetirom-treated patients with MASH cirrhosis (mean change from baseline with 95% CI; p value for comparison vs. zero) by the presence of MRI-PDFF response.

| **Year 1 Week 52** | MRI-PDFF response present | p | MRI-PDFF response absent | p | P b/w groups |
| --- | --- | --- | --- | --- | --- |
| Abdominal symptoms | -0.21 (-0.51-0.09) | 0.25 | 0.13 (-0.30-0.56) | 0.81 | 0.26 |
| Activity/energy | -0.14 (-0.38-0.11) | 0.23 | 0.01 (-0.43-0.45) | 0.63 | 0.56 |
| Emotional function | 0.01 (-0.21-0.24) | 0.74 | 0.29 (-0.11-0.69) | 0.18 | 0.23 |
| Fatigue | -0.02 (-0.30-0.25) | 0.50 | 0.15 (-0.22-0.52) | 0.39 | 0.52 |
| Systemic symptoms | -0.13 (-0.32-0.06) | 0.22 | 0.01 (-0.24-0.25) | 0.85 | 0.46 |
| Worry | 0.20 (-0.07-0.48) | 0.0414 | 0.51 (-0.02-1.04) | 0.12 | 0.29 |
| Total CLDQ-NASH score | -0.05 (-0.22-0.13) | 0.64 | 0.18 (-0.07-0.44) | 0.41 | 0.19 |
| Physical Functioning (PF) | -0.21 (-4.75-4.34) | 0.82 | -0.71 (-12.34-10.91) | 0.73 | 0.92 |
| Role Physical (RP) | -0.38 (-4.83-4.06) | 0.76 | 0.60 (-9.55-10.74) | 1.00 | 0.84 |
| Bodily Pain (BP) | -4.48 (-9.88-0.92) | 0.08 | -0.29 (-7.76-7.19) | 0.79 | 0.43 |
| General Health (GH) | -1.63 (-5.09-1.83) | 0.21 | 1.67 (-3.07-6.41) | 0.46 | 0.34 |
| Vitality (VT) | -0.58 (-4.12-2.96) | 0.93 | 0.30 (-6.54-7.14) | 0.93 | 0.82 |
| Social Functioning (SF) | -0.96 (-5.72-3.79) | 0.59 | 7.74 (-2.47-17.95) | 0.21 | 0.10 |
| Role Emotional (RE) | 3.08 (-0.84-6.99) | 0.11 | 2.38 (-11.24-16.01) | 0.79 | 0.90 |
| Mental Health (MH) | -0.15 (-3.59-3.28) | 0.70 | -1.19 (-7.27-4.89) | 0.67 | 0.77 |
| Physical Component Summary (PCS) | -1.08 (-2.98-0.82) | 0.35 | 0.05 (-3.13-3.23) | 0.96 | 0.56 |
| Mental Component Summary (MCS) | 0.57 (-1.24-2.39) | 0.42 | 1.03 (-2.79-4.85) | 0.93 | 0.82 |
| Symptoms of liver disease | -0.92 (-4.19-2.35) | 0.71 | -2.22 (-7.25-2.80) | 0.47 | 0.69 |
| Effects of liver disease | -0.72 (-4.13-2.70) | 0.39 | 0.32 (-5.90-6.53) | 0.92 | 0.77 |
| Concentration/memory | -1.15 (-5.04-2.73) | 0.84 | 0.00 (-6.44-6.44) | 0.73 | 0.77 |
| Health distress | 4.42 (-1.09-9.94) | 0.09 | 5.36 (-8.12-18.84) | 0.59 | 0.88 |
| Sexual Function | 1.37 (-12.92-15.66) | 0.59 | -1.39 (-20.74-17.96) | 0.56 | 0.84 |
| Sleep | -1.46 (-4.95-2.03) | 0.33 | 2.38 (-3.46-8.22) | 0.70 | 0.28 |
| Loneliness | -0.55 (-5.18-4.09) | 0.88 | -5.00 (-9.43- -0.57) | 0.0333 | 0.31 |
| Hopelessness | -2.05 (-7.07-2.96) | 0.98 | -7.54 (-14.84- -0.24) | 0.06 | 0.27 |
| **Stigma of liver disease** | **3.94 (0.07-7.81)** | **0.09** | **-4.17 (-10.45-2.12)** | **0.27** | **0.0419** |
| Total LDQOL | 0.24 (-2.07-2.56) | 0.60 | -1.45 (-5.32-2.43) | 0.51 | 0.48 |
| **Year 2 Week 52** | MRI-PDFF response present | p | MRI-PDFF response absent | p | P b/w groups |
| Abdominal symptoms | 0.25 (-0.13-0.63) | 0.29 | -0.11 (-0.54-0.33) | 0.69 | 0.26 |
| Activity/energy | -0.09 (-0.42-0.23) | 0.32 | -0.28 (-0.61-0.05) | 0.08 | 0.48 |
| Emotional function | -0.06 (-0.31-0.18) | 0.51 | 0.04 (-0.35-0.44) | 0.76 | 0.64 |
| Fatigue | -0.06 (-0.40-0.28) | 0.73 | -0.01 (-0.46-0.43) | 0.92 | 0.86 |
| Systemic symptoms | -0.12 (-0.38-0.14) | 0.38 | 0.01 (-0.31-0.32) | 0.72 | 0.56 |
| Worry | 0.02 (-0.37-0.42) | 0.90 | 0.15 (-0.40-0.71) | 0.63 | 0.70 |
| Total CLDQ-NASH score | -0.01 (-0.25-0.23) | 0.79 | -0.03 (-0.27-0.20) | 0.82 | 0.91 |
| Physical Functioning (PF) | -2.47 (-6.94-2.01) | 0.15 | -4.80 (-12.06-2.46) | 0.15 | 0.58 |
| Role Physical (RP) | -2.16 (-6.81-2.48) | 0.25 | -1.50 (-8.62-5.62) | 0.73 | 0.88 |
| Bodily Pain (BP) | -2.42 (-8.58-3.74) | 0.35 | 2.16 (-5.57-9.89) | 0.77 | 0.39 |
| General Health (GH) | -4.37 (-8.64- -0.09) | 0.05 | -1.84 (-8.04-4.36) | 0.52 | 0.51 |
| Vitality (VT) | -0.84 (-5.22-3.53) | 0.64 | -1.50 (-8.29-5.29) | 0.78 | 0.87 |
| Social Functioning (SF) | 0.48 (-5.15-6.11) | 0.99 | -3.50 (-13.15-6.15) | 0.60 | 0.46 |
| Role Emotional (RE) | -2.40 (-7.87-3.06) | 0.47 | 1.00 (-7.76-9.76) | 0.72 | 0.50 |
| Mental Health (MH) | -3.85 (-7.85-0.16) | 0.0376 | 0.00 (-7.18-7.18) | 0.99 | 0.33 |
| Physical Component Summary (PCS) | -0.88 (-2.83-1.08) | 0.52 | -1.00 (-2.86-0.86) | 0.27 | 0.94 |
| Mental Component Summary (MCS) | -1.02 (-3.14-1.11) | 0.76 | 0.05 (-3.46-3.57) | 0.95 | 0.59 |
| Symptoms of liver disease | -1.73 (-5.28-1.81) | 0.30 | 0.13 (-6.10-6.37) | 0.93 | 0.59 |
| Effects of liver disease | 0.90 (-3.05-4.85) | 0.60 | -6.40 (-12.27- -0.53) | 0.0375 | 0.0444 |
| Concentration/memory | -4.45 (-8.76- -0.13) | 0.0476 | 2.25 (-2.79-7.29) | 0.51 | 0.07 |
| Health distress | 5.53 (-2.47-13.53) | 0.15 | 1.00 (-6.20-8.20) | 0.77 | 0.48 |
| Sexual Function | -8.02 (-24.96-8.92) | 0.39 | 1.46 (-15.44-18.36) | 0.95 | 0.50 |
| Sleep | -0.58 (-4.99-3.83) | 0.86 | 0.20 (-6.60-7.00) | 0.81 | 0.85 |
| **Loneliness** | **4.62 (0.82-8.41)** | **0.0079** | **-4.40 (-10.45-1.65)** | **0.20** | **0.0124** |
| Hopelessness | -5.45 (-10.30- -0.60) | 0.06 | -0.33 (-8.58-7.91) | 0.96 | 0.27 |
| Stigma of liver disease | -0.36 (-6.09-5.37) | 0.85 | -8.50 (-15.24- -1.76) | 0.0200 | 0.10 |
| Total LDQOL | -0.30 (-2.82-2.21) | 0.30 | -1.83 (-5.38-1.72) | 0.55 | 0.50 |

**Supplementary Table 11**. Changes in HRQL scores in resmetirom-treated patients with MASH cirrhosis (mean change from baseline with 95% CI; p value for comparison vs. zero) by the presence of VCTE response.

| **Year 1 Week 52** | VCTE response present | p | VCTE response absent | p | P b/w groups |
| --- | --- | --- | --- | --- | --- |
| Abdominal symptoms | -0.21 (-0.55-0.14) | 0.19 | 0.20 (-0.07-0.46) | 0.25 | 0.07 |
| Activity/energy | -0.12 (-0.40-0.15) | 0.26 | -0.08 (-0.33-0.17) | 0.39 | 0.82 |
| Emotional function | 0.02 (-0.24-0.27) | 0.53 | 0.06 (-0.13-0.25) | 0.86 | 0.77 |
| Fatigue | -0.02 (-0.30-0.27) | 0.67 | -0.12 (-0.35-0.12) | 0.33 | 0.59 |
| Systemic symptoms | -0.03 (-0.24-0.18) | 0.89 | -0.20 (-0.37- -0.03) | 0.0253 | 0.20 |
| Worry | 0.17 (-0.16-0.51) | 0.24 | 0.38 (0.14-0.62) | 0.0009 | 0.33 |
| Total CLDQ-NASH score | -0.03 (-0.25-0.19) | 0.78 | 0.04 (-0.11-0.19) | 0.60 | 0.60 |
| Physical Functioning (PF) | 2.03 (-3.30-7.37) | 0.50 | -0.19 (-4.99-4.61) | 0.87 | 0.54 |
| Role Physical (RP) | 2.05 (-3.59-7.69) | 0.54 | 1.50 (-3.25-6.24) | 0.75 | 0.88 |
| Bodily Pain (BP) | 0.28 (-5.20-5.76) | 0.89 | -3.70 (-8.13-0.73) | 0.11 | 0.27 |
| General Health (GH) | -1.47 (-5.38-2.44) | 0.43 | -1.63 (-4.32-1.05) | 0.37 | 0.94 |
| Vitality (VT) | -0.39 (-4.36-3.58) | 0.97 | -1.06 (-5.05-2.94) | 0.62 | 0.82 |
| Social Functioning (SF) | 0.98 (-4.96-6.91) | 0.98 | 1.58 (-2.84-6.01) | 0.36 | 0.87 |
| Role Emotional (RE) | 1.95 (-3.84-7.75) | 0.21 | 3.17 (-1.77-8.11) | 0.18 | 0.75 |
| Mental Health (MH) | 1.09 (-2.99-5.18) | 0.77 | -2.04 (-5.09-1.00) | 0.13 | 0.22 |
| Physical Component Summary (PCS) | 0.16 (-1.89-2.21) | 0.80 | -0.55 (-2.00-0.90) | 0.75 | 0.58 |
| Mental Component Summary (MCS) | 0.42 (-1.76-2.60) | 0.33 | 0.22 (-1.58-2.01) | 0.76 | 0.88 |
| Symptoms of liver disease | -0.10 (-3.58-3.38) | 0.97 | -1.27 (-3.82-1.29) | 0.52 | 0.59 |
| Effects of liver disease | -3.85 (-7.87-0.17) | 0.0493 | -1.50 (-4.48-1.48) | 0.20 | 0.35 |
| Concentration/memory | -2.44 (-6.61-1.73) | 0.27 | -1.50 (-4.96-1.97) | 0.36 | 0.73 |
| Health distress | 4.49 (-2.37-11.36) | 0.21 | 2.99 (-0.98-6.97) | 0.10 | 0.71 |
| Sexual Function | -2.40 (-12.35-7.56) | 0.89 | 1.76 (-12.15-15.67) | 0.47 | 0.64 |
| Sleep | -2.11 (-5.63-1.41) | 0.18 | 0.07 (-3.52-3.66) | 0.69 | 0.40 |
| Loneliness | 0.32 (-4.65-5.28) | 0.79 | -0.42 (-4.22-3.38) | 0.40 | 0.81 |
| Hopelessness | -3.78 (-8.97-1.42) | 0.60 | 2.11 (-2.69-6.91) | 0.52 | 0.10 |
| Stigma of liver disease | 1.37 (-3.14-5.87) | 0.86 | 1.14 (-2.65-4.94) | 0.50 | 0.94 |
| Total LDQOL | -0.92 (-3.47-1.62) | 0.66 | 0.28 (-1.67-2.24) | 0.95 | 0.46 |
| **Year 2 Week 52** | VCTE response present | p | VCTE response absent | p | P b/w groups |
| Abdominal symptoms | 0.14 (-0.20-0.48) | 0.46 | 0.05 (-0.40-0.51) | 0.66 | 0.76 |
| Activity/energy | 0.00 (-0.29-0.29) | 0.40 | -0.20 (-0.54-0.15) | 0.28 | 0.39 |
| Emotional function | -0.10 (-0.36-0.17) | 0.25 | 0.11 (-0.15-0.37) | 0.41 | 0.27 |
| Fatigue | -0.08 (-0.41-0.25) | 0.84 | 0.03 (-0.28-0.34) | 0.81 | 0.63 |
| Systemic symptoms | -0.15 (-0.40-0.11) | 0.23 | -0.05 (-0.29-0.20) | 0.89 | 0.58 |
| Worry | 0.32 (-0.03-0.67) | 0.12 | -0.04 (-0.46-0.39) | 0.83 | 0.20 |
| Total CLDQ-NASH score | 0.02 (-0.19-0.24) | 0.92 | -0.01 (-0.24-0.22) | 0.55 | 0.82 |
| Physical Functioning (PF) | -2.29 (-7.49-2.90) | 0.29 | -2.57 (-6.64-1.50) | 0.13 | 0.93 |
| Role Physical (RP) | 0.00 (-5.32-5.32) | 0.93 | 1.17 (-4.10-6.44) | 0.47 | 0.76 |
| Bodily Pain (BP) | 0.77 (-5.75-7.29) | 0.91 | -1.02 (-6.43-4.39) | 0.54 | 0.68 |
| General Health (GH) | -2.02 (-6.21-2.17) | 0.44 | -4.85 (-9.67- -0.04) | 0.0522 | 0.39 |
| Vitality (VT) | -1.04 (-6.28-4.20) | 0.75 | -2.34 (-6.51-1.82) | 0.35 | 0.70 |
| Social Functioning (SF) | 1.04 (-5.14-7.22) | 0.91 | -2.86 (-8.85-3.12) | 0.52 | 0.38 |
| Role Emotional (RE) | -0.17 (-6.02-5.67) | 0.66 | -2.08 (-7.64-3.47) | 0.42 | 0.64 |
| Mental Health (MH) | -1.98 (-7.61-3.65) | 0.31 | -2.19 (-5.88-1.50) | 0.20 | 0.95 |
| Physical Component Summary (PCS) | -0.29 (-2.31-1.73) | 0.77 | -0.58 (-2.09-0.93) | 0.74 | 0.82 |
| Mental Component Summary (MCS) | -0.35 (-2.92-2.22) | 0.86 | -1.25 (-3.34-0.83) | 0.20 | 0.59 |
| Symptoms of liver disease | 0.07 (-4.22-4.36) | 0.85 | -4.03 (-8.12-0.06) | 0.0516 | 0.18 |
| Effects of liver disease | -2.22 (-6.94-2.50) | 0.26 | -1.25 (-4.83-2.33) | 0.77 | 0.75 |
| Concentration/memory | -3.78 (-8.49-0.93) | 0.09 | 0.00 (-4.33-4.33) | 0.62 | 0.25 |
| **Health distress** | 9.90 (2.75-17.04) | 0.0090 | -1.82 (-8.55-4.90) | 0.70 | 0.0214 |
| Sexual Function | -0.78 (-15.36-13.80) | 0.96 | -3.33 (-16.06-9.39) | 0.98 | 0.80 |
| Sleep | -1.46 (-5.51-2.59) | 0.28 | 1.67 (-3.32-6.66) | 0.41 | 0.34 |
| Loneliness | 2.40 (-2.17-6.96) | 0.23 | 1.35 (-2.32-5.03) | 0.41 | 0.73 |
| Hopelessness | -0.17 (-5.17-4.82) | 0.97 | -5.90 (-11.61- -0.19) | 0.0268 | 0.14 |
| Stigma of liver disease | -1.17 (-6.29-3.94) | 0.85 | -1.04 (-6.88-4.80) | 0.95 | 0.97 |
| Total LDQOL | 0.39 (-2.26-3.04) | 0.90 | -1.31 (-3.69-1.07) | 0.31 | 0.35 |

**Supplementary Figure 1**. Changes in HRQL scores in patients with early MASH with (Responder) vs. without MRI-PDFF (≥30%) response (Nonresponder) to treatment with resmetirom (pooled 100 mg and 80 mg) vs. placebo; mean HRQL score change from baseline to Week 52 with 95% CI. (A) CLDQ-NAFLD scores range 1-7; (B) LDQOL scores range 0-100.


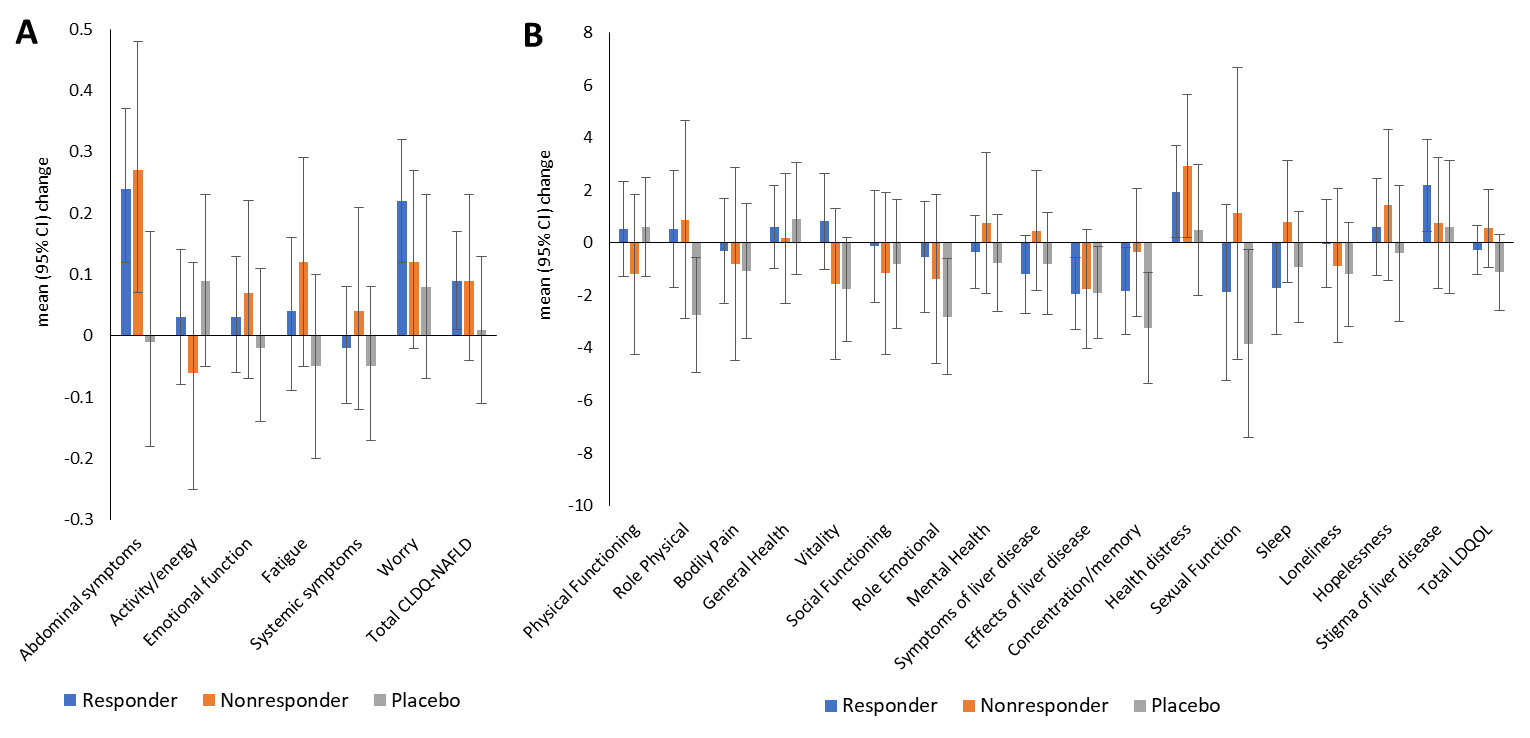

Supplement: SUPPLEMENTARY MATERIAL [file hc9-10-e00913-s001.docx]
